# Phenazine Cations as Anticancer Theranostics†

**DOI:** 10.1021/jacs.4c03491

**Published:** 2024-04-29

**Authors:** Felicity F. Noakes, Kirsty L. Smitten, Laura E. C. Maple, Jorge Bernardino de la Serna, Craig C. Robertson, Dylan Pritchard, Simon D. Fairbanks, Julia A. Weinstein, Carl G. W. Smythe, Jim A. Thomas

**Affiliations:** ‡ Department of Chemistry, The University of Sheffield, Western Bank, Sheffield S3 7HF, U.K.; § Department of Biomedical Science, The University of Sheffield, Western Bank, Sheffield S10 2TN, U.K.; ∥ Department of Molecular Biology and Biotechnology, The University of Sheffield, Western Bank, Sheffield S10 2TN, U.K.; ⊥ National Heart and Lung Institute, Imperial College London, London SW7 2AZ, U.K.; # Central Laser Facility, Rutherford Appleton Laboratory, Research Complex at Harwell, Science and Technology Facilities Council, Harwell-Oxford, Didcot OX11 0QX, U.K.

## Abstract

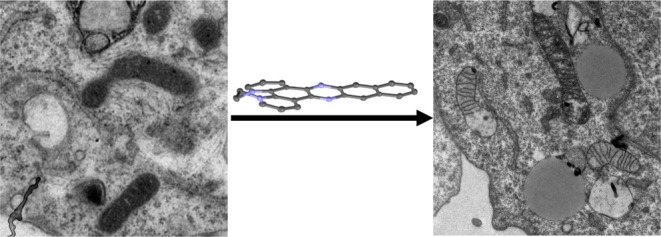

The biological properties of two water-soluble organic cations based on polypyridyl structures commonly used as ligands for photoactive transition metal complexes designed to interact with biomolecules are investigated. A cytotoxicity screen employing a small panel of cell lines reveals that both cations show cytotoxicity toward cancer cells but show reduced cytotoxicity to noncancerous HEK293 cells with the more extended system being notably more active. Although it is not a singlet oxygen sensitizer, the more active cation also displayed enhanced potency on irradiation with visible light, making it active at nanomolar concentrations. Using the intrinsic luminescence of the cations, their cellular uptake was investigated in more detail, revealing that the active compound is more readily internalized than its less lipophilic analogue. Colocalization studies with established cell probes reveal that the active cation predominantly localizes within lysosomes and that irradiation leads to the disruption of mitochondrial structure and function. Stimulated emission depletion (STED) nanoscopy and transmission electron microscopy (TEM) imaging reveal that treatment results in distinct lysosomal swelling and extensive cellular vacuolization. Further imaging-based studies confirm that treatment with the active cation induces lysosomal membrane permeabilization, which triggers lysosome-dependent cell-death due to both necrosis and caspase-dependent apoptosis. A preliminary toxicity screen in the *Galleria melonella* animal model was carried out on both cations and revealed no detectable toxicity up to concentrations of 80 mg/kg. Taken together, these studies indicate that this class of synthetically easy-to-access photoactive compounds offers potential as novel therapeutic leads.

## Introduction

Luminescent ruthenium(II) polypyridyl complexes containing coordinated dppz and dppn ligands are well documented as cellular imaging agents and anticancer therapeutics.^1−12^ And in this context, we have reported on a number of dinuclear complexes incorporating such ligands as potential phototherapeutics and theranostics.^13−16^ However, the biological properties of water-soluble, metal-free derivatives of these DNA intercalating ligands are considerably less studied.^17^ We have reported that cationic derivatives of these compounds, such as **1**^2+^ and **2**^2+^, bind to DNA with affinities comparable to many metal complexes and are capable of photo-oxidizing DNA in cell-free studies.^18−20^ Elmes et al. reported that a related system based on an electron deficient ligand is cell permeant, localizes in the cytoplasm, and—although not intrinsically cytotoxic—displays significant phototoxicity through the generation of ROS.^21^

Herein, we report on the cellular uptake, localization, imaging properties, cytotoxicity, and phototoxicity of **1**^2+^ and **2**^2+^. These studies reveal that one of these compounds displays activities comparable to established anticancer therapeutics and is active in therapeutically resistant cancer cells. Furthermore, its emission properties mean that its cellular uptake and localization can be visualized through super-resolution optical microscopy techniques.

## Results and Discussion

**Figure 1:**
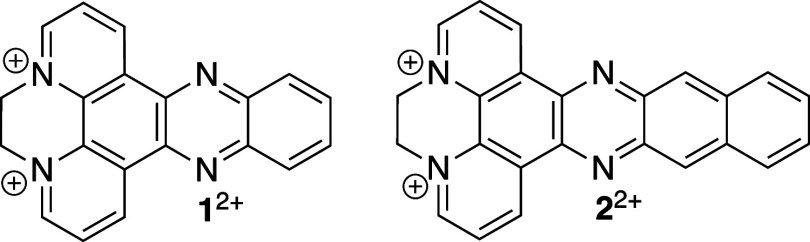
Chemical structures of dqDPPZ, 1^2+^ and dqDPPN, 2^2+^.

Two diquaternized cations with extended aromatic systems were chosen to be investigated. Dipyrido [3,2-a:2′,3′-c]phenazine (dqDPPZ) **1**^2+^ and benzodipyrido[a:3,2-h:29,39-*j*]phenazine (dqDPPN) **2**^2+^ were synthesized according to reported methods (Figure 1).^18,19^

### X-ray Crystallographic Studies

We previously reported the crystal structures of dppz and **[1]**(PF_6_)_2_^18^ and the structure of dppn has recently been described;^22^ however, the structure of **[2]**(PF_6_)_2_ has yet to be reported. In our new studies, we found X-ray quality crystals of this compound could be obtained by vapor diffusion of diethyl ether into nitromethane solutions.

**Figure 2:**
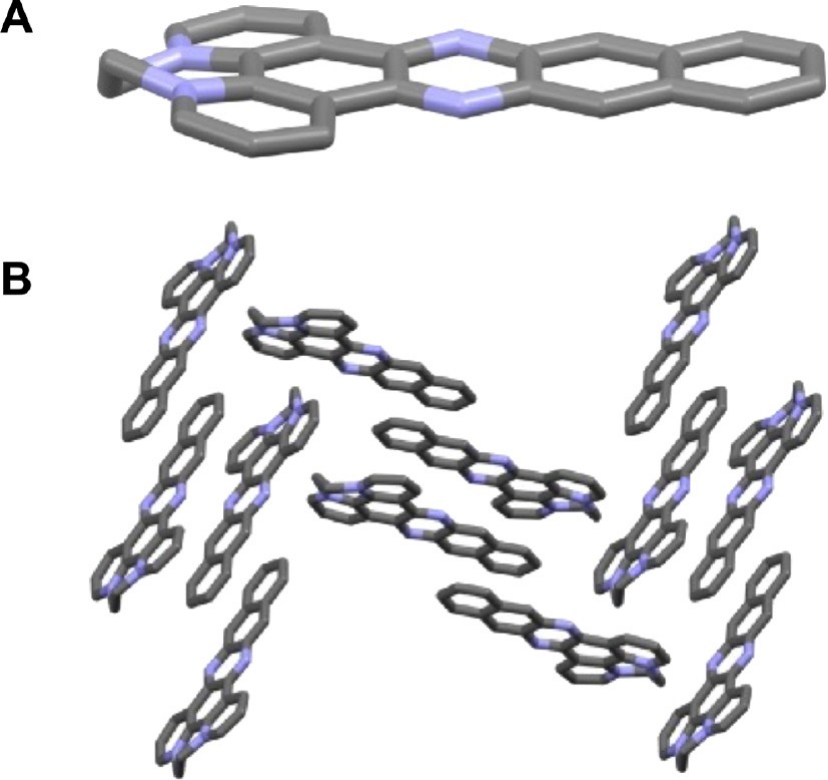
Details are from crystallographic structures of [2](PF_6_)_2_. (A) The structure of the 2^2+^ cations. (B) Packing of the 2^2+^ units revealing head-to-tail offset stacking of the aromatic systems. Hexafluorophosphate counterions have been removed for ease of visualization of this packing.

The crystal structure of **[2]**[PF_6_] shows that the cation twists to accommodate the ethylene bridge, thus its dppn unit deviates from complete planarity (Figure 2a). To accommodate the charge of the dicationic units, a herringbone pattern in which dppn moieties are stacked in alternating orientation is observed, with anions filling spaces between these aromatic stacks (Figure 2b). Further crystallographic data on this structure are shown in the Supporting Information (see SI, Section S3a).

### Cell Studies

Previous reports on [**1**]Cl_2_ and [**2**]Cl_2_ have demonstrated that they are water-soluble and entirely stable in aqueous solutions,^18,19^ given these properties their interactions with live cells were investigated. To gain some preliminary insights into whether **1**^2+^ and **2**^2+^ could be cell permeant, log* P* values for both compounds were determined using the octanol and water shake-flask method. These measurements confirmed that both cations are lipophilic (see SI, S3b) and, as might be expected from its more extended aromatic system, **2**^2+^ is more lipophilic than **1**^2+^.

#### Cytotoxicity

**Figure 3:**
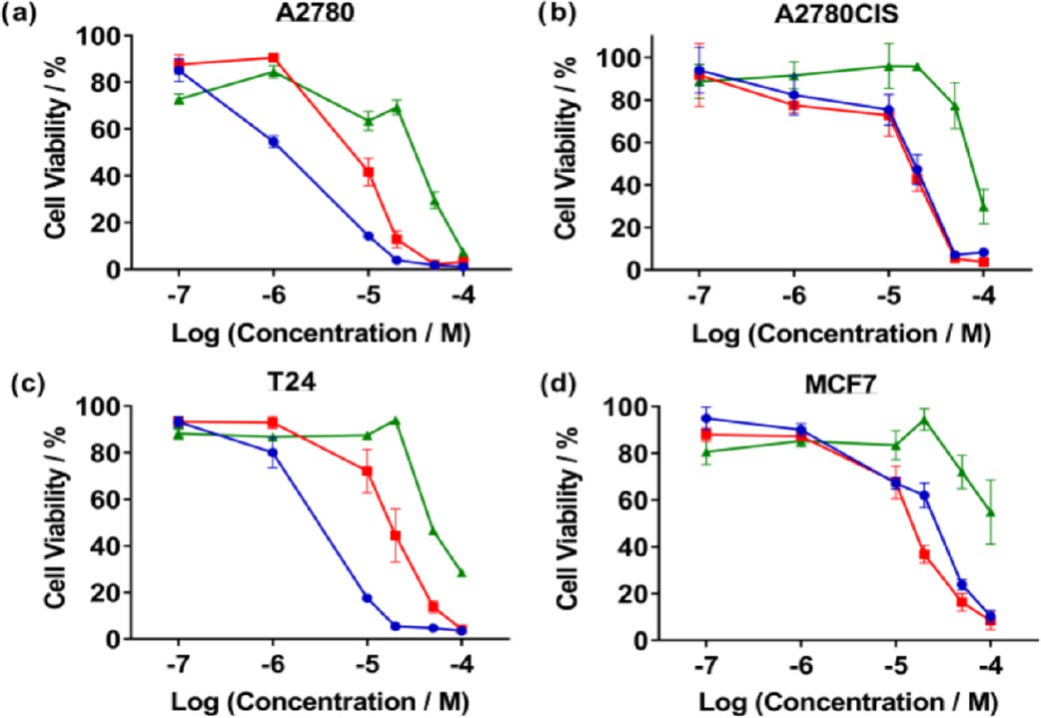
Cell viability data for (A) A2780, (B) A2780CIS, (C) T24, and (D) MCF7 cell lines after treatment with each compound for 48 h analyzed by an MTT assay. Cisplatin was employed as a positive control. The experiments were carried out in triplicate and are given as an average of three biological replicates. Green = [1]Cl2; Red = [2]Cl2; Blue = cisplatin.

First, the cytotoxicity of **1**^2+^ and **2**^2+^ toward several cancer cell lines was examined. The viability of cells exposed to their chloride salts was assessed by an MTT (3-(4,5-dimethylthiazol-2-yl)-2,5-diphenyltetrazolium bromide) assay, and the common chemotherapeutic agent cisplatin was employed as a positive control, Figure 3.

Estimated IC_50_ values obtained through these experiments are summarized in Table 1. Interestingly, **2**^2+^ exhibits the highest potency in the human ovarian carcinoma cell line A2780, and its potency is only slightly lower in the cisplatin-resistant daughter cell line A2780CIS, with a resistance factor (RF) of ∼2 being observed, indicating minimal cross-resistance. Given this promising result, both **1**^2+^ and **2**^2+^ were tested in further cancer cell lines in comparison to cisplatin.

**Table 1:** IC_50_ Values for Compounds [1]Cl_2_ and [2]Cl_2_ in Selected Cell Lines in Comparison to Cisplatin

	IC_50_ values μM (S.D.) in specified cell lines				
compound	A2780	A2780CIS	T24	MCF7	HEK293
[1]Cl2	32(4)	74(9)	48(2)	≥100	86(7)
[2]Cl2	8(3)	17(3)	18(6)	15(2)	23(5)
cisplatin	1.3(1)	19(3)	3(1)	26(2)	5(2)

The HER2-positive, MCF7 breast carcinoma cell line showed even more promising results. Although **1**^2+^ displays minimal effects on this line (IC_50_ = ∼100 μM), **2**^2+^ was found to be more cytotoxic (IC_50_ = 15 μM) than cisplatin (IC_50_ = 26 μM). However, with the T24 bladder carcinoma cell line, both cations showed lower potency compared to cisplatin; for example, **2**^2+^ showed a toxicity of 18 μM compared to a IC_50_ of 3 μM for cisplatin. Nevertheless, with all four cell lines, **2**^2+^ displays a higher potency than **1**^2+^ and yet it also shows reduced cytotoxicity to noncancerous HEK293 cells (Table 1, see SI Section S3c for data) and, significantly, it is much less toxic to this line than cisplatin.

#### Phototoxicity

Previous metal complexes of these types of ligands have shown potential application as photodynamic therapy (PDT) agents.^4,8,12,23−31^ Given the promising dark IC_50_ values detailed above, the cytotoxicity of **1**^2+^ and **2**^2+^ on exposure to light was also investigated. Consequently, the A2780 and A2780CIS cell lines were treated with concentration gradients of [**1**]Cl_2_ and [**2**]Cl_2_ and exposed to different light fluences.

**Figure 4:**
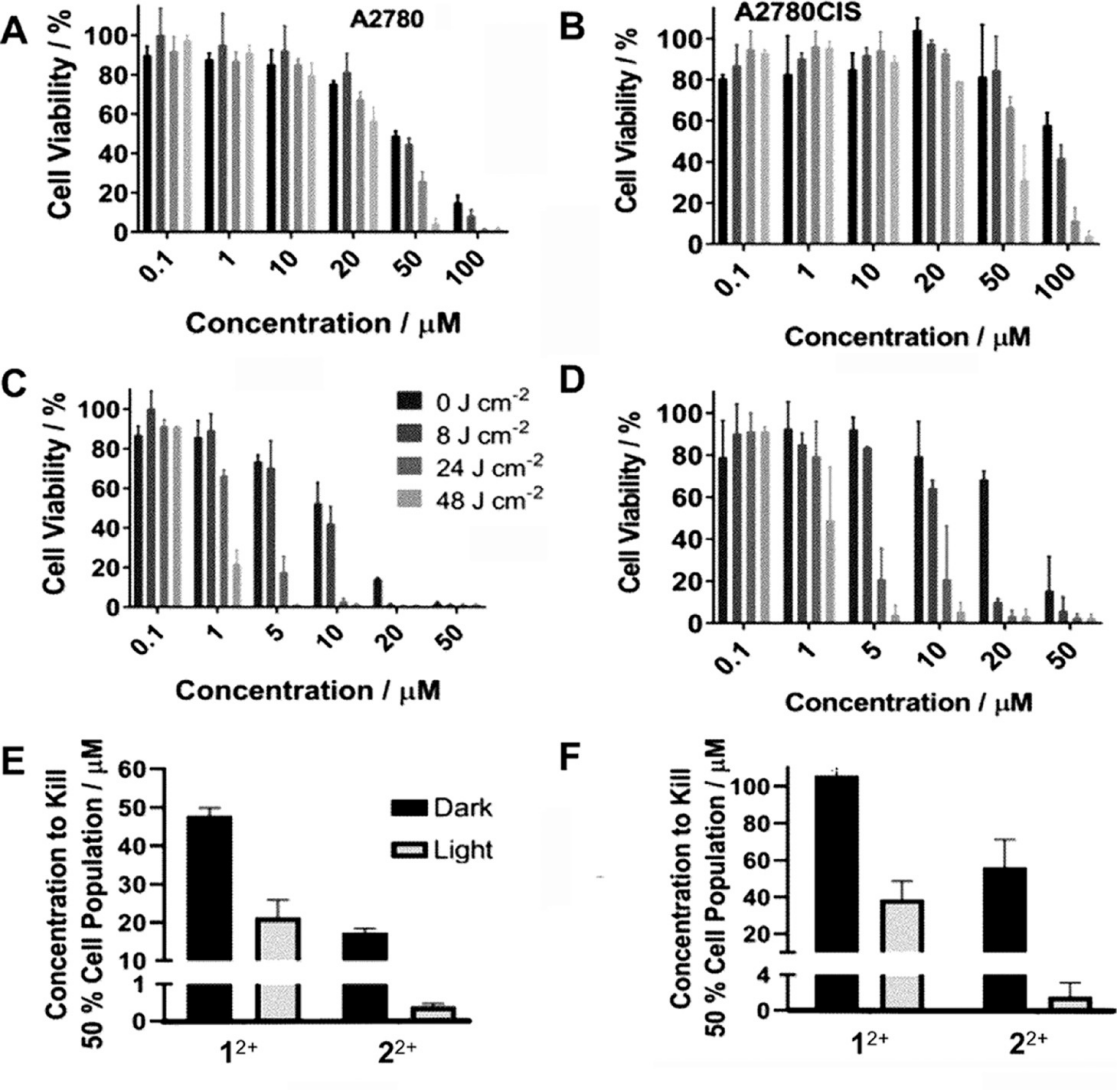
Light and dark cell viability graphs after treatment with varying concentrations of [1]Cl_2_ and [2]Cl_2_ with changing light fluences. Experiments were performed in triplicate with three biological replicates. (A) A2780 cells with treatment at varying concentrations of 1. (B) A2780CIS cells with treatment of 1. (C) A2780 cells with treatment of 2. (D) A2780CIS cells with treatment of 2. (E) Comparison of light and dark IC_50_ values for 1 and 2 in A2780 cells. (F) Comparison of light and dark IC_50_ values for both compounds in A2780CIS cells.

These studies revealed that both compounds show enhanced potency under light irradiation. As shown in Figure 4, the cells are susceptible to both the concentration of cations and light dosage. However, while **1**^2+^ only shows a modest enhancement in phototoxicity (see SI, S3d), **2**^2+^ demonstrates a dramatic decrease in IC_50_ in both cell lines and a phototoxicity index, PI (PI = IC_50_Dark/IC_50_Light), of 42. In the cisplatin-resistant line, this figure is even higher (PI = 54) Table 2. Thus, with only a relatively moderate light fluence of 48 J cm^–2^, the IC_50_ of **2**^2+^ is decreased to nanomolar concentrations (390 nM) in A2780 cells. Even in A2780CIS cells, the IC_50_ of **2**^2+^ is 1 μM, rendering it approximately 20 times more active than cisplatin in this therapeutically resistant line. Given these results, the possibility that the compounds produce singlet oxygen was then explored.

**Table 2:** IC_50_ Values of 2^2+^ Toward the A2780 and A2780CIS Cell Lines in the Presence and Absence of Light

	IC_50_ values μM (S.D.) in specific cell lines	
fluence (J cm^–2^)	A2780	A2780CIS
0	17(1)	54(15)
8	13(3)	27(3)
24	2(0.5)	8.9(2)
48	0.4(0.8)	1(1.5)

The singlet oxygen quantum yields for each compound were obtained from the hexafluorophosphate salts dissolved in acetonitrile by directly measuring the fluorescence intensity of ^1^O_2_ at 1270 nm after excitation at 355 nm. A standard of perinaphthenone with a singlet oxygen quantum yield of 1.0 in acetonitrile was employed as a reference sensitizer for comparison. The Φ(^1^O_2_) values obtained for **1**^2+^ and **2**^2+^ using this procedure were 0.354 ± 0.02 and 0, respectively, revealing that the most photoactive cation does not generate ^1^O_2_.

This result supports our previously published cell-free studies, revealing that the high-energy excited state of both cations can directly photo-oxidize DNA;^18,19^ thus, the enhanced photocytotoxicity of **2**^2+^ within cell can be attributed to direct photodamage, unmediated by ^1^O_2_. Contrastingly, although **1**^2+^ is both directly photo-oxidizing and a moderate singlet oxygen sensitizer, it is significantly less active than **2**^2+^ in the dark or when illuminated. Taken together, these observations suggest that the cellular uptake and localization of the two cations is different. We then exploited their intrinsic luminescence to investigate this question.

#### Cell Imaging Studies

Cells were treated with the cations for 24 h and any exocellular compound was removed prior to imaging using an enhanced laser scanning confocal microscopy technique, Airyscan, which provides subdiffraction lateral resolutions to 120 nm.^32−34^ These studies indicate that although compound **1**^2+^ is taken up by live cells, as its characteristic luminescence centered on 510 nm is observed in the cytoplasm, the emission is very weak (see SI, S3e) and unevenly distributed within the cytoplasm. Taken together with its relatively low cytotoxicity and phototoxicity, these observations indicate that **1**^2+^ is poorly taken up by live cells.

**Figure 5:**
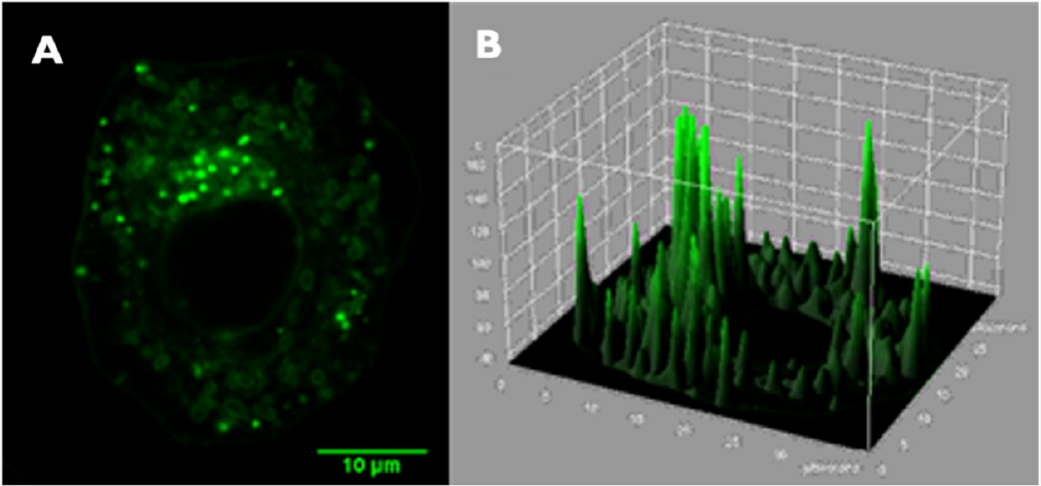
(A) Live cell image of A2780 cells following 24 h exposure to 10 μM of 2^2+^; (B) 3D profile plot of image A.

In contrast, images of the more lipophilic **2**^2+^ cation in both A2780 cells (Figure 5) and MCF7 cells (see SI, S3e) reveal bright intracellular emission throughout the cytoplasm but not the nucleus. The uptake process is energy dependent as virtually no internalization is observed at reduced temperatures, observations which are consistent with endocytosis. To further probe the intracellular target of **2**^2+^, colocalization experiments with commercially available stains were employed.

#### Colocalization Studies

As expected from the preliminary images, costaining with a nuclear stain (DRAQ5) and endoplasmic reticulum trackers revealed little to no overlay (See SI, Section Sf). Given the punctate emission seen in the cytoplasm, the possibility that **2**^2+^ localizes in lysosomes or mitochondria was investigated.

**Figure 6:**
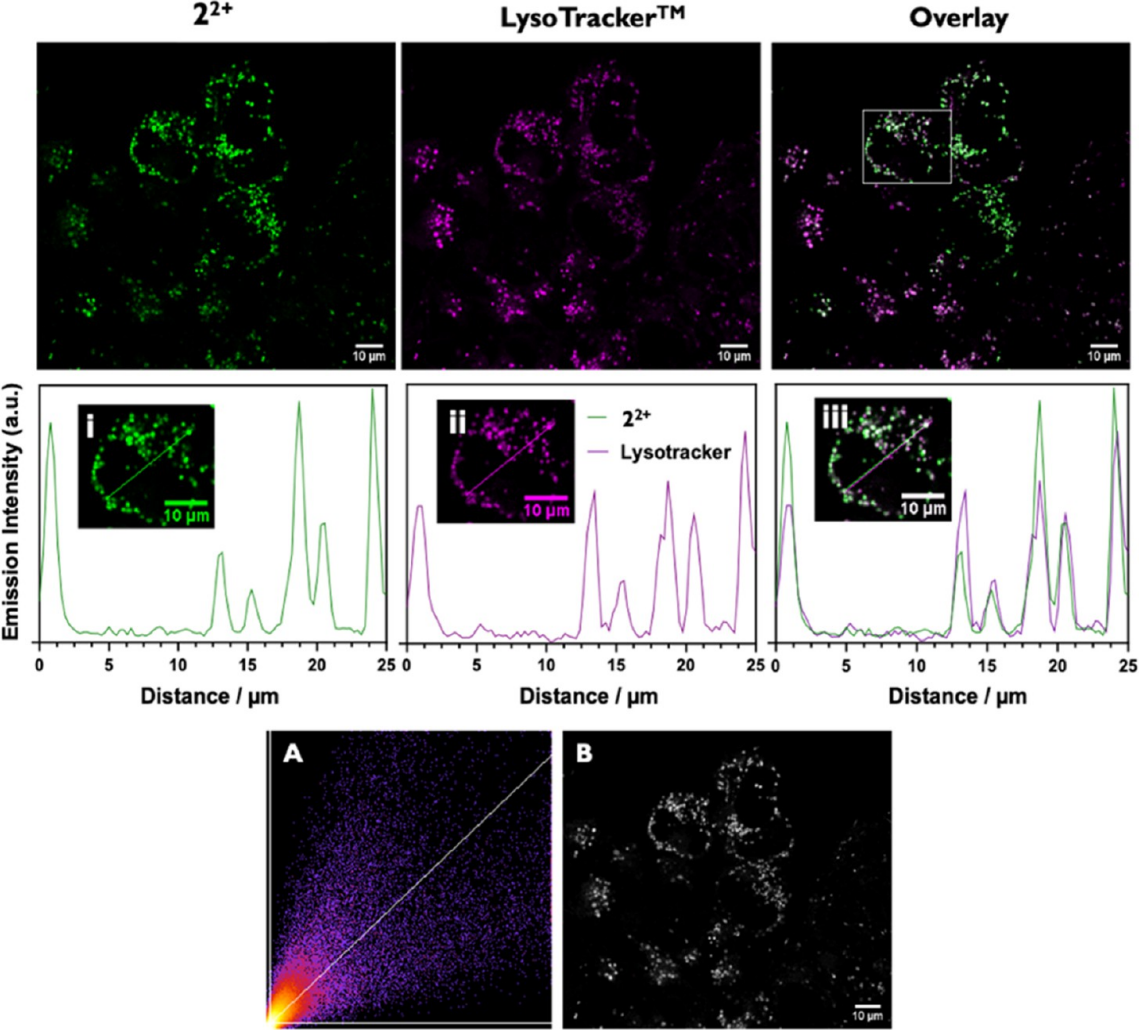
Top: Colocalization images of A2780 cells were stained with 10 μM 2^2+^ and LysoTracker Deep Red. Luminescence emission of 2^2+^ (green), LysoTracker Deep Red (magenta), and overlay. Middle: Distance vs emission profiles of single cell. Bottom: Emission signals as (A) scatter plot; (B) intensity map where overlapping signals are shown in white.

The commercially available dye LysoTracker Deep Red (LTDR) was employed as a costain to investigate lysosomal uptake. LTDR absorbs at 644 nm and emits at 668 nm in the deep red channel, whereas **2**^2+^ absorbs at 410 nm and emits in the green channel, thus avoiding any overlap of emission. As the images in Figure 6 illustrate, both **2**^2+^ and LTDR appear to possess a common intracellular location. The calculated Pearson’s coefficient of 0.781 confirms and quantifies this overlay and is indicative of high colocalization; furthermore, plots of distance against intensity provide further evidence for this conclusion.

**Figure 7:**
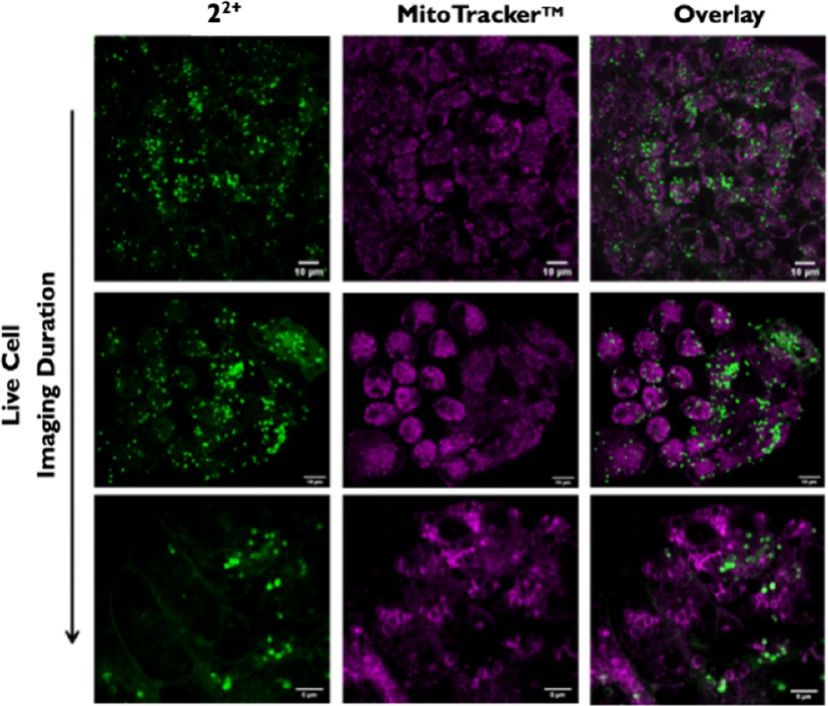
Live cell imaging of A2780 cells treated with 10 μM [2]Cl_2_ for 6 h followed by MTDR to observe colocalization and phototherapeutic effect. From top to bottom: 0 to 1 min of live cell imaging shows vacuolization of mitochondria on irrradiation.

As cationic lipophilic structures often localize in mitochondria,^14,35−38^ this possibility was also investigated. In this case, cells were costained with a commercial MitoTracker and, again, the deep red variant, MTDR, was chosen to avoid emission overlap. As the images in Figure 7 show, there was minimal colocalization between **2**^2+^ and MTDR, confirming that lysosomes are the main target for this cation.

#### Effects on Mitochondria Morphology and Function

MTDR costaining was revealing in another way, as it allowed the phototoxic effects of **2**^2+^ to be directly observed. In real-time live cell imaging experiments, mitochondria were seen to swell and then form vacuolar structures, Figure 7, lower row (See also SI, Section S3g). These observations indicate that mitochondrial dysfunction has a role in the phototherapeutic effect of **2**^2+^. Further evidence for this hypothesis comes from the observation that during these live cell experiments, the Pearson’s coefficient for **2**^2+^ and MTDR increases from 0.25 to 0.43, indicating that **2**^2+^ internalizes within mitochondria after illumination through membrane disruption.

Given the effects on mitochondria observed through microscopy, a TMRE (tetramethylrhodamine, ethyl ester) assay was used to investigate whether treatment of A2780 cells with **2**^2+^ induces a loss of mitochondrial membrane potential. FCCP (carbonyl cyanide 4-(trifluoromethoxy) phenylhydrazone), which causes depolarization of mitochondrial membranes, was employed as a positive control. Even without exposure to light, overnight treatment of **2**^2+^ at the IC_50_ caused a decrease in the mitochondrial membrane potential, and this effect is enhanced after light exposure (See SI, S3h) suggesting that, both in the dark and light, **2**^2+^ induces mitochondrial dysfunction.

#### Using STED Nanoscopy to Probe Effects on Lysosomes

Although the application of **2**^2+^ as a cellular imaging probe is limited due to the potent phototoxicity, its potential compatibility with fixed-cell STED nanoscopy was investigated as this technique can provide resolutions down to 20 nm.^39−41^

**Figure 8:**
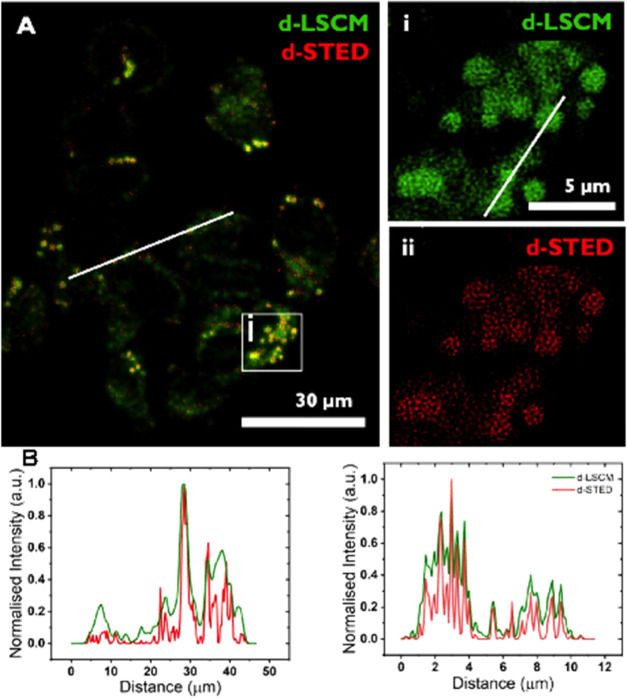
(A) A2780 cells treated with 10 μM of [2]Cl_2_. (i) Comparison of CLSM (top right) and (ii) STED (lower right) of the white box shown in the main image. (B) comparison of CLSM and STED intensity profiles across the white lines drawn on the left and right images shown in panel (A).

Surprisingly, clear super-resolution images of lysosomes in cells labeled with **2**^2+^ could be obtained—Figure 8. The enhanced resolution obtained using this technique is demonstrated by a comparison of intensity plots for lysosomal luminescence obtained from STED and Airyscan.

Using STED, planes taken through treated cells and 3D reconstruction reveal in remarkable detail that treatment with **2**^2+^ also results in distinctively enlarged lysosomal structures, confirming that the cation is internalized within lysosomes and revealing that—apart from its effect on mitochondria—the cation also alters the morphology of lysosomes as well (see SI, S3i).

#### Electron Microscopy Reveals 2^2+^ Causes Extensive Vacuolization

To gain further insight into the therapeutic effects of **2**^2+^, transmission electron microscopy (TEM) was employed to image cells after treatment under dark conditions and when illuminated. Given the vacuolization observed after light exposure, we sought to confirm that mitochondrial and lysosomal damage caused by **2**^2+^ was potentiated by light exposure.

**Figure 9:**
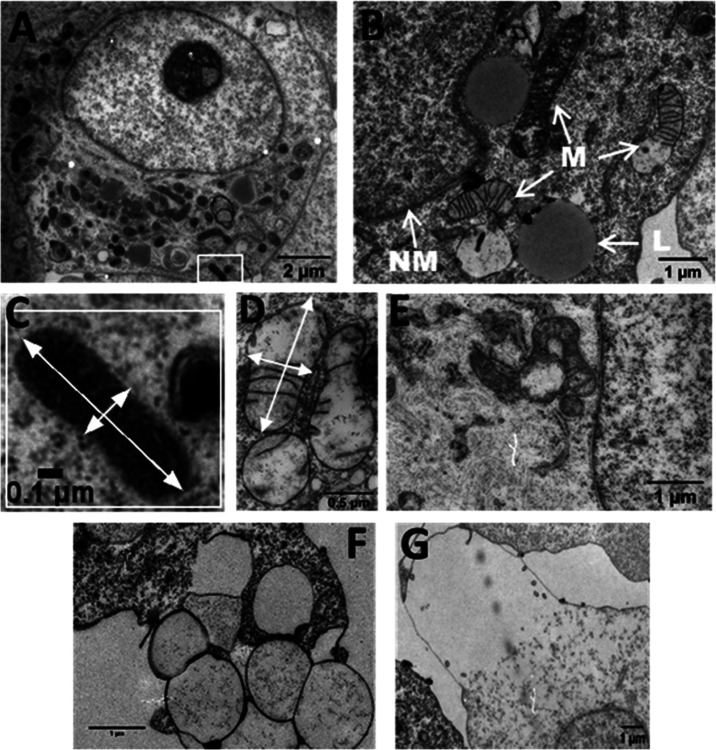
TEM of A2780 cells; (A) control without compound; (B) treatment with 50 μM 2^2+^ for 24 h; (C) a mitochondrion from the control; (D) swollen mitochondria after treatment with 50 μM of 2^2+^ for 24 h; (E) mitochondria after treatment with 8 μM of 2^2+^ for 24 h; (F) vacuoles after treatment with 8 μM of 2^2+^ for 24 h; (G) swollen membrane after treatment with 8 μM of cation for 24 h. Labels NM = nuclear membrane, M = mitochondria, and L = lysosomes.

First, A2780 cells were treated with **2**^2+^ at and above the IC_50_ overnight without illumination and then fixed and processed for imaging. Although controls showed compact mitochondria with a well-defined inner membrane forming the cristae at both treatment concentrations, mitochondria displayed significant structural damage, Figure 9. While treatment at the IC_50_ concentration resulted in detectable mitochondrial swelling and some loss of internal structure, at the higher treatment concentration, mitochondria were greatly enlarged with almost complete loss of internal structure such as cristae—Figure 9A,B.

A typical mitochondrion in the untreated control measured a width of 0.23 μm and a length of 0.82 μm (Figure 9C), but typical post-treatment measurements were significantly larger at 0.63 μm by 1.4 μm (Figure 9D).

In addition to mitochondrial enlargement, EM also revealed the extensive formation of vacuoles (Figure 9f). Optical microscopy and TEM studies after illumination were also carried out, and these revealed similar effects but even more extreme vacuolization cell swelling, and eventually, loss of membrane integrity with cell membrane bursting (see SI, Section S3j).

Extensive vacuolization is a characteristic of several cell death mechanisms, including paraptosis, autophagy, and necrosis;^42−44^ however, further studies provided evidence that treatment with **2**^2+^ leads to distinctive lysosomal changes that are key to the cell death it induces.

#### Exposure to 2^2+^ Causes Lysosomal Membrane Permeabilization

The live cell microscopy experiments described above clearly reveal lysosomal swelling, and although colocalization studies with LysoTracker confirmed that **2**^2+^ accumulates in lysosomes, at later time points, there was visibly less puncta due to lysosomal staining and those that remained had increased in size (Figure 8 and SI, S3ik), suggesting that **2**^2+^ induces lysosomal membrane permeabilization (LMP), an effect that is enhanced or initiated by light.^45,46^ Just as mitochondrial outer membrane permeabilization is known as a key event in apoptosis,^47^ LMP acts to trigger lysosome-dependent cell-death, LDCD, a process that is known to cause extensive vacuolization and also leads to mitochondrial damage and disfunction.^48−50^ LMP, which triggers release of lysosomal contents such as cathepsins and hydrolases into the cytosol, can be caused by the generation of ROS.^48,51^ Alternatively, amines which are capable of protonating inside lysosomes can act as a lysosomotropic agent.^48,52^ As **2**^2+^ also bears a structural resemblance to such lysosomal detergents, the possibility that it induces LMP was investigated through two assays.

First, a galectin puncta assay was employed. This method is based on the translocations of small sugar binding proteins, galectins, to damaged lysosomes.^53,54^ This results in a characteristic punctate staining pattern, as the glycolipid-coated inner surface of the lysosomal membrane becomes accessible for binding. Galectin accumulation to permeabilized lysosomes can then be observed through immunofluorescence with a galectin-3 antibody.^55^

In these experiments, A2780 cells were exposed to 20 μM **2**^2+^ prior to fixation by PFA at an early and late time point. To provide a comparison to any phototoxic effects, cells were also treated with 5 μM **2**^2+^ for 24 h followed by 10 min of light irradiation prior to fixation and processing with galectin antibodies. As a positive control, cells were incubated with LLOMe, a lysosomal disruption agent^56^ that is known to increase galectin puncta.

**Figure 10:**
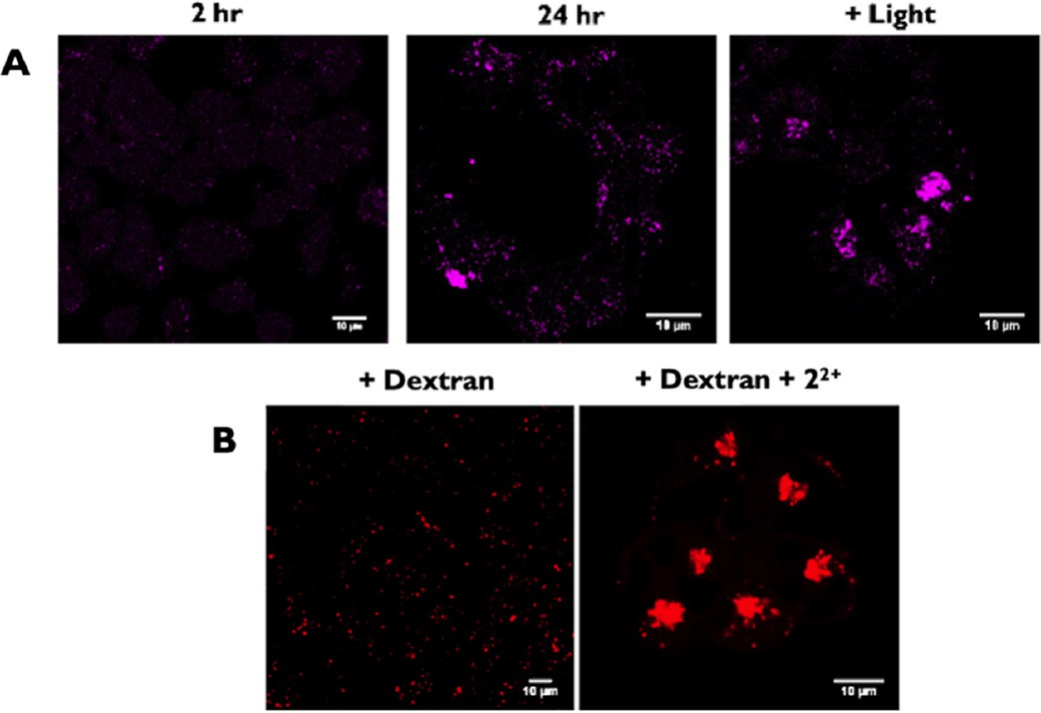
(A) Galectin puncta formation through immunofluorescence assay. A2780 cells were exposed to 20 μM 2^2+^ at two different time points or 5 μM followed by 10 min of light irradiation. Cells were fixed in 4% PFA and stained with an anti-LGALS3 antibody. (B*)* Dextran release assay. A2780 cells were preloaded with fluorescent dextran prior to exposure for 7 h. Left: cells loaded with dextran only; right: cells loaded with dextran and treated with 2^2+^.

Treatment with **2**^2+^ increased the galectin puncta in comparison to untreated control—Figure 10A. The later 24 h time point shows an increase in puncta compared to the earlier 2 h time point which was comparable to that caused by LLOMe (see SI, Section S3l), indicating that lysosomal integrity was damaged after treatment with **2**^2+^. LMP was investigated through a second method involving the release of a fluorescent tetramethylrhodamine conjugated dextran into the cytosol.^57^

By taking advantage of endocytosis, lysosomes were loaded with fluorescent dextran prior to treatment with **2**^2+^. The effect of LMP was then detected by monitoring translocation of dextran from lysosomes into the cytosol. In uncompromised negative controls, fluorescent dextran remains localized in lysosomes and is visible as punctate structures within cells, whereas after LMP, this transitions to a more diffuse cytosolic staining. A2780 cells were preloaded with fluorescent dextran and exposed to 20 μM **2**^2+^ for 7 h. As expected from the galectin-based experiments, after treatment, the puncta observed in the control are no longer apparent, Figure 10B, confirming the hypothesis that **2**^2+^ induces LMP.

#### Cell Death Signaling

It is known that LDCD can induce more than one death mechanism; in particular, cathepsins released by LMP can trigger necrosis and classical caspase-dependent apoptosis.^48,50,58,59^ So, although morphological changes observed in cells treated with **2**^2+^ are consistent with necrosis (a known consequence of extensive LMP), early loss of mitochondrial membrane potential is also observed in apoptotic cell death; therefore, this issue was investigated in more detail.

To further understand how **2**^2+^ induces cell death, we treated A2780 cells with varying concentrations of **2**^2+^ with and without a brief period of light irradiation. We used live and fixed-cell flow cytometry to investigate cell morphology, integrity, DNA fragmentation, and molecular markers associated with cell death mechanisms.^60,61^ Apoptosis is characterized by the progressive emergence of a cell population in which dying cells are substantially reduced in size.^60^ Apoptotic cell shrinkage is accompanied by cell-surface expression of phosphatidyl-serine (PS), and followed by progressive DNA fragmentation.^61^ In contrast, necrotic cell death is characterized by very rapid changes in cell size, accompanied by dramatic increases in granularity (as measured by flow cytometric side-scatter), reflecting rapid nuclear and cytoplasmic condensation.^60^ Necrotic cells can also display PS staining, coupled with significant loss in cell integrity.^60,62^ PS^+^ staining may reflect the cell-surface expression of PS or the increased accessibility of impermeant PS stain to the interior of cells whose plasma membrane is compromised.

**Figure 11:**
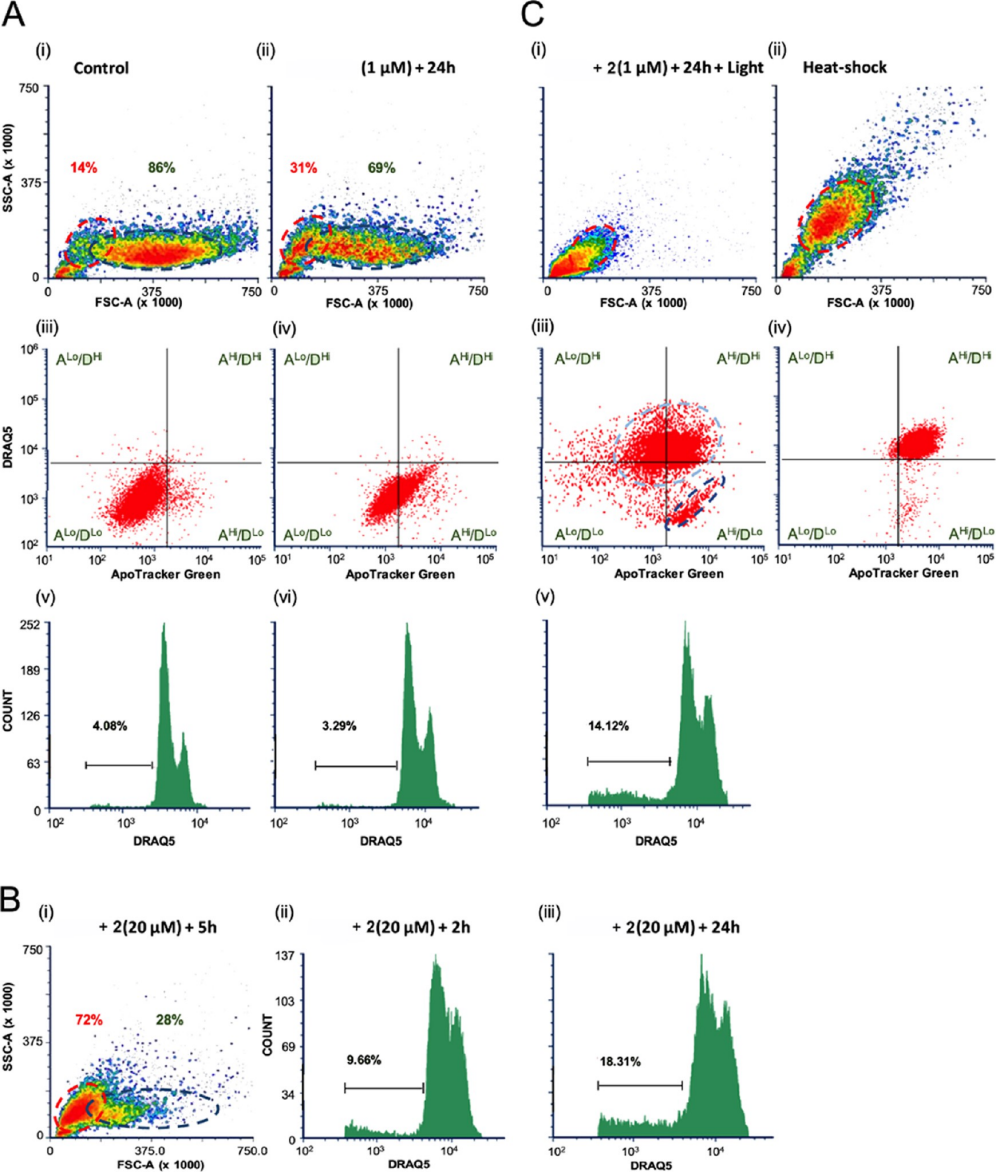
Flow cytometric analyses of A2780 cells following 2^2+^, dqDPPN, treatment in the presence and absence of light. Cells were exposed to the indicated concentrations of 2^2+^ for various times and subsequent light irradiation. Cells were then either (A, C: (i–iv), B: (i)) exposed to DRAQ5 (10 μM) and ApoTracker Green (200 nM) and subjected to live cell flow cytometry or (A, C: bottom panels, B: (ii),(iii)) fixed and stained with DRAQ5 (5 μM) for DNA content.

When compared with controls (Figure 11A(i)), treatment with a low dose (1 μM) of **2**^2+^ for 24 h resulted in the appearance of a population of small cells (Figure 11A(ii), upper panel, red oval) as indicated by reduced forward scatter, with no significant change in side-scatter. Treated cells largely excluded the DNA dye DRAQ5 (D^lo^ segments) and displayed an increased expression of PS as determined by ApoTracker staining (A^Hi^ segments, Figure 11A(iii)). These data strongly suggest that, after 24 h, cells treated with low-dose **2**^2+^ display early signs of apoptosis. Consistent with an early apoptotic phenotype,^62,63^ fixed-cell flow cytometry of the same cells showed no increase in cells with a sub-G1 DNA content (Figure 11A(vi)). Exposure of cells to high-dose (20 μM) **2**^2+^ resulted in a faster emergence of apoptotic cells with most live cells (72%) displaying substantially reduced forward scatter (Figure 11B(i)) with no DRAQ5 uptake (see SI SXB) within 5 h of treatment. Analysis of fixed cells previously exposed to high-dose **2**^2+^ for both shorter (2 h) and longer (24 h) durations showed substantial increase in populations of cells with sub-G1 DNA content, rising from ∼4% in control cells to 18% in cells treated for 24 h. This was accompanied by a parallel loss of G2 DNA content in the same cell population, again consistent with apoptotic cell death (Figure 11B(ii),(iii)).

Transient light irradiation and a further 1 h incubation after treatment with low-dose (1 μM) **2**^2+^ for 24 h gave rise to a very rapid cell swelling phase (see SI, S3j) followed by fast and comprehensive size reduction as observed by reduced forward- and side-scatter measurements (Figure 11C(i), red oval). In this population of live cells, there was substantial loss of cell integrity as judged by a significant increase in DRAQ5 uptake (D^Hi^ segments) (Figure 11C(iii), ligh blue oval), together with a significant increase in accessible PS in the majority of cells (Figure 11C(iii), A^Hi^/D^Hi^). However, a substantial proportion of this cell population (D^Hi^) displayed DRAQ5 uptake but not high levels of ApoTracker staining (Figure 11C(iii), A^Lo^/D^Hi^), indicating that plasma membrane integrity was compromised only in these cells, without any increase in PS accessibility. Taken together, these data indicate that the combination of light and **2**^2+^ results in the rapid onset of a form of necrotic cell death, characterized by loss of plasma membrane integrity, with a broad range of levels of accessible PS.

Oncosis, a form of necrotic cell death, is characterized by mitochondrial dysfunction and vacuolization as observed in Figures 7 and 9. A population of A2780 cells was subjected to heat-shock to induce oncosis,^64^ for cytometric comparison with light and **2**^2+^-treated cells. When exposed to DRAQ5 and ApoTracker, heat-shocked cells displayed loss of cell integrity (Figure 11C(iv), D^Hi^) as well as elevated levels of accessible PS (Figure 11C(iv), A^Hi^), resembling, in part, the distribution observed in light and **2**^2+^-treated cells. However, in contrast, heat-shocked cells displayed a very substantial increase in granularity, evidenced by increased side-scatter, with limited reduction in cell size (Figure 11C(ii), red oval), differing significantly with those observed with light irradiation and **2**^2+^ treatment consistent with the distinct morphologies observed under light microscopy (data not shown). The data suggest that cell death induced by a combination of light irradiation and exposure to **2**^2+^ is distinct from oncosis, although the possibility that treatment reflects a rapid transition to a late stage of oncosis cannot be completely ruled out.

Importantly, a distinct small population of cells (indicated by the dark blue oval, Figure 11C(iii)) that underwent the same treatment retained cell integrity (D^Lo^ segments), while displaying elevated levels of cell-surface PS as measured by ApoTracker (A^Hi^/D^Lo^). In fixed cells, flow cytometry showed a significant increase from ∼4 to ∼14% of cells with a sub-G1 DNA content as well as cells with a sub-G2 content (Figure 11C(v)), indicating that they have undergone an accelerated transition to later stages of apoptosis. It is possible that this population may have committed to apoptosis prior to light exposure.

Taken together with observations made above, these data support the notion that **2**^2+^ induces apoptosis in cells in the absence of light, and that the added irradiation results in the rapid onset of one or more forms of necrosis, with characteristics of vacuolization, organelle, and cell swelling, observed in various forms of necrotic cell death, in addition to a degree of continued apoptosis.^65^ These data confirm that more than one pathway is involved in **2**^2+^-induced cell death, whereby both apoptosis and necrosis are triggered by LMP.

#### *Galleria melonella*

Given the encouraging activity of **2**^2+^, a preliminary toxicity screen in an animal model was carried out. Larvae of the wax moth *G. melonella* exhibit physiology aspects, such as body temperature and immune system,^66,67^ which are very similar to mammals. Consequently, they are increasingly employed as an *in vivo* model, including as a toxicity screen, yielding results that are comparable to commonly used mammalian models.^68−72^ Indeed, recent studies comparing *Galleria* to rodents demonstrate their effectiveness in bridging the gap between *in vitro* studies and animal models for toxicity screening.^71,73−76^

**Figure 12:**
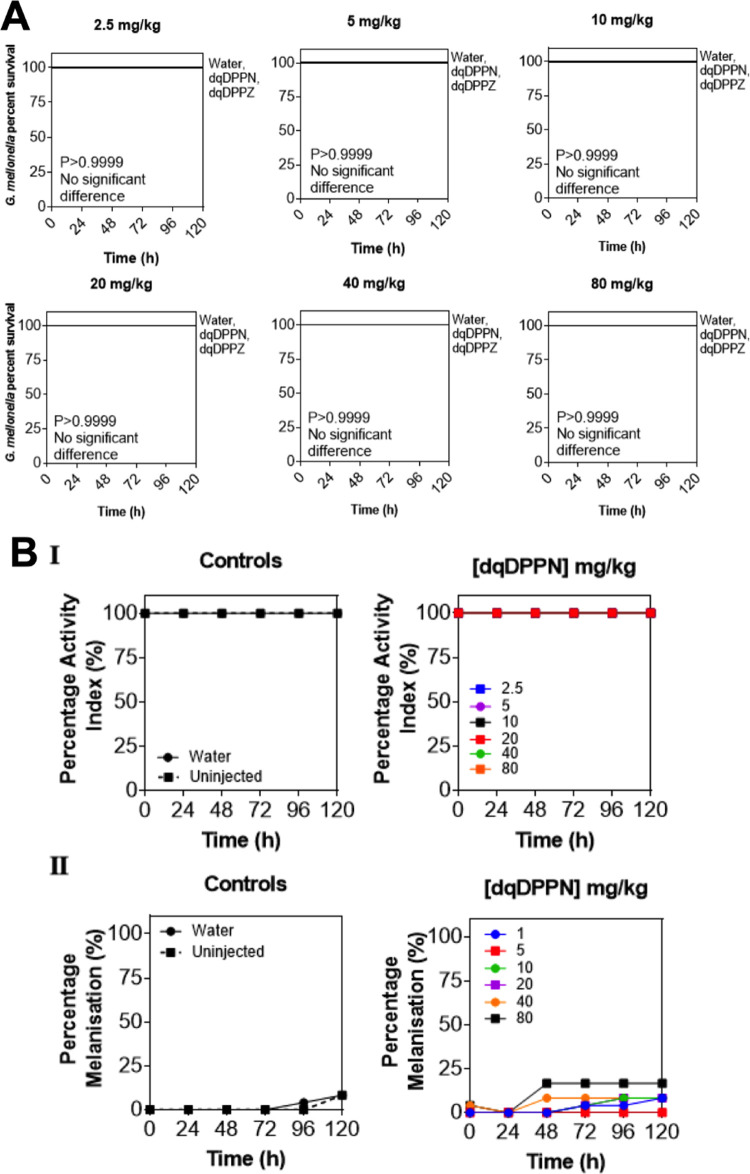
*G. melonella* toxicity screen data for 2^2+^ (A) Kaplan–Meier survival curves after treatment at concentrations ranging 0–80 mg/kg. Kept at room temperature and monitored over a period of 120 h. (B(i)) Activity scores of the larvae obtained every 24 h after exposure. (B(ii)) Melanisation levels recorded every 24 h for 120 h and plotted as a percentage.

The toxicity screen was carried out on both **1**^2+^ and **2**^2+^ with the larvae monitored over a period of 120 h at room temperature and treated with doses up to 80 mg/kg. The compounds were dissolved in water and injected through the last left proleg using a Hamilton syringe, and results were plotted as Kaplan–Meier survival graphs. The results for **2**^2+^ are shown in Figure 12 (see SI Section S3m for the equivalent data on **1**^2+^).

Often for infection models, the larvae are kept at the optimum temperature for bacterial survival of 37 °C which is equivalent to human body temperature; however, this was not necessary for a toxicity screen. Nevertheless, a comparative study at this temperature, which is suboptimal for the larvae, was also carried out. As might be expected, increased melanization and a lower survival rate in both the control and treated population was observed at the higher temperature (see SI).

Although some minor melanization was observed in the treated larvae, with **2**^2+^ inducing a slightly larger effect than **1**^**2+**^, neither **1**^2+^ nor **2**^2+^ produced any significant negative effects on the treated *Galleria* compared to controls, confirming that at the concentrations employed, both compounds showed little to no toxicity effects on larvae.

## Conclusions

There are a huge number of reports on the biological activity of transition metal complexes containing extended phenanthroline-type ligands designed to interact with biomolecules. Many of these studies have revealed fascinating effects that have been exploited in the construction of probes, therapeutics and phototherapeutics, and theranostics. However, it seems likely that commercial exploitation and bulk availability of specific pharmaceuticals based on platinum group metals will be hampered by the low Earth abundance of raw materials and questions about the metabolic fate of these abiotic elements within the body. Herein, we demonstrate that structurally related, simple to synthesize, organic cations based on these ligand systems can display potent therapeutic and phototherapeutic effects in themselves.

Significantly, we have shown that **2**^2+^ is active against a range of cancer lines, being particularly active against the therapeutically resistant A2780CIS ovarian cancer line and the aggressive HER2-positive MCF7 breast cancer line. As this cation triggers LMF, leading to cell death through more than one death mechanism, it seems likely that it will be active against a range of therapeutically resistant cancers that often abrogate apoptotic signaling responses. Given these results and the ease of synthetic access to this class of compounds, studies on even more difficult-to-treat cancers are being developed and will form the basis of future reports.

## Supporting Information Available

Chemical methods, tissue culture, microscopy and *in vivo* methods, summary of crystallographic data and structure refinement, cell viability graph for HEK293 cells, phototoxicity values, live cell imaging, partition coefficients, mitochondrial membrane potential assay, costain microscopy with DRAQ5 and ER-Tracker, live cell imaging of phototoxicity, electron microscopy, further LMP microscopy details, additional cytometry, *Galleria Mellonella* Kaplain-Meier survival curves at 37 °C, instrumentation (PDF)
