# Supplementary material for: Phenazine Cations as Anticancer Theranostics†
Source: J Am Chem Soc. 2024 Apr 29;146(18):12836–49. doi: 10.1021/jacs.4c03491 (PMC11082890; doi:10.1021/jacs.4c03491)
Supplement: Supplementary file 1 — ja4c03491_si_001.pdf [file ja4c03491_si_001.pdf]

Supporting Information

Phenazine Cations as Anticancer Theranostics

Felicity F. Noakes,[a,b] Kirsty L. Smitten,[a,c] Laura E.C. Maple,[b] Jorge Bernardino de la Serna,[d,e] Craig C. Robertson,[a] Dylan Pritchard,[a] Simon D. Fairbanks,[a] Julia A Weinstein,[a] Carl G.W. Smythe,[*b] Jim A. Thomas[*a]

[a]Department of Chemistry, The University of Sheffield, Western Bank, Sheffield, U.K.

[b]Department of Biomedical Science, The University of Sheffield, Western Bank, Sheffield, S10 2TN, U.K.

[c]Department of Molecular Biology and Biotechnology, The University of Sheffield, Western Bank, Sheffield, S10 2TN, U.K.

[d]National Heart and Lung Institute, Imperial College London, SW7 2AZ, UK.

[e]Central Laser Facility, Rutherford Appleton Laboratory, Research Complex at Harwell, Science and Technology Facilities Council, Harwell-Oxford, Didcot OX11 0QX, U.K

[a]Department of Chemistry, The University of Sheffield, Western Bank, Sheffield, U.K.

[b]Department of Biomedical Science, The University of Sheffield, Western Bank, Sheffield, S10 2TN, U.K.

[c]Department of Molecular Biology and Biotechnology, The University of Sheffield, Western Bank, Sheffield, S10 2TN, U.K.

[d]National Heart and Lung Institute, Imperial College London, SW7 2AZ, UK.

[e]Central Laser Facility, Rutherford Appleton Laboratory, Research Complex at Harwell, Science and Technology Facilities Council, Harwell-Oxford, Didcot OX11 0QX, U.K

Table of Contents

S1. Chemical Methods
S1a. Anion Metathesis
S1b. Singlet Oxygen Quantum Yield Determination
S1c. Partition Coefficient Determination
S1d. X-Ray Crystallography

S2. Tissue Culture, Microscopy, and *in vivo* Methods
S2a. Cell Culture
S2b. Preparation of Compounds
S2c. Cytotoxicity (MTT Assay) and Determination of IC_{50} Values
S2d. Phototoxicity
S2e. Mitochondrial Membrane Potential Assay (TMRE Assay)
S2f. Single Stain Live Cell Microscopy
S2g. LysoTracker® Deep Red Co-stain Microscopy
S2h. Mitotracker Co-stain Microscopy
S2i. DRAQ5 Co-Stain Microscopy
S2j. ER-Tracker Co-Stain Microscopy
S2k. Single Stain Fixed Cell Microscopy
S2l. Transmission Electron Microscopy
S2m. Cytometry
S2n. *Galleria Mellonella* Toxicity Screen

S3. Supplementary Data
S3a. Summary of Crystallographic Data and Structure Refinement
S3b.Partition Coefficients
S3c. Cell Viability Graph for HEK293 Cells

S3d. Phototoxicity Values
S3e. Live Cell Imaging
S3f. Costain Microscopy with DRAQ5 and ER-Tracker
S3g. Live Cell Imaging of Phototoxicity and Mitotracker Controls
S3h. Mitochondrial Membrane Potential Assay
S3i. Single cell STED Imaging revealing enlarged lysosomal structures
S3j. Electron Microscopy
S3k.Lysosomal Quantification

S3l. Galectin Controls
S3m Cytometry – additional data
S3m. *Galleria Mellonella* Kaplain-Meier Survival Curves at 37 ^{o}C

S4. Instrumentation
S4a. Microscopy
S4b. Photochemistry
S4c. Electron Microscopy
S4d. NMR Spectroscopy
S4e. X-Ray Crystallography

S1. Chemical Methods

Compounds **1** - **2** were synthesized using previously reported methods.

S1a. Anion Metathesis

Compounds were converted from hexafluorophosphate salts to chloride salts by stirring over a DOWEX anion-exchange resin in distilled water. The DOWEX was then removed by filtration and the filtrate dried *in vacuo* to yield either $\mathbf{1}^{2+}$ or $\mathbf{2}^{2+}$ as a chloride salt.

S1b. Singlet Oxygen Quantum Yield Determination

Compounds $\mathbf{1}^{2+}$ and $\mathbf{2}^{2+}$ were dissolved in acetonitrile as hexafluorophosphate salts and the optical densities adjusted to 0.1 at 355 nm. The samples were then irradiated with a short pulse Nd-YAG laser at 355 nm. Since singlet oxygen fluoresces at 1270 nm, an NIR detector was used at 77 K that measures fluorescence intensity throughout the pulse. This fluorescence intensity was then measured at various pulse powers starting at 20 μJ through to 500 μJ. The measurement is an average of 512 readings taken by the oscilloscope, and an average of five readings was recorded at each power. The amplitudes of the fluorescence at each power were then compared to peerinaphthenone which has a singlet oxygen quantum yield of 100 % in acetonitrile, to give a quantum yield at each power which was then averaged out.

S1c. Determination of Log *P*

Octanol-water partition coefficients were obtained via the shake-flask method to determine the relative lipophilicity of $\mathbf{1}^{2+}$ and $\mathbf{2}^{2+}$. Aqueous stocks of a known concentration were prepared and added to an equal volume of 1-octanol that had been previously saturated with water overnight. After 24 hours with shaking, each phase was recovered and the concentration of compound in each layer was determined by UV-vis absorption spectroscopy.

S1d. X-Ray Crystallography

X-ray quality crystals of the quaternised hexafluorophosphate salt **2** were obtained by vapour diffusion using a concentrated solution in nitromethane with diethyl ether as an antisolvent.

Data for $\mathbf{2}(PF_6)_2$ were collected on a Bruker Kappa Apex-II CCD diffractometer utilising a MoKα sealed-tube X-ray source. Reflections were corrected for absorption by empirical methods (SADABS) based upon symmetry equivalent reflections in combination with measurements at varied azimuthal angles.[1,2] The crystal structures were determined and refined against F2 values using ShelXT for solution and ShelXL for refinement through the Olex2 program.[3,4] Hydrogen atoms were positioned according to calculations with ideal geometries and refined utilising a riding model and isotropic displacement parameters. Non-hydrogen atoms were refined anisotropically.

S2. Tissue Culture and Microscopy Methods

S2a. Cell Culture

Cell media was purchased sterile from Sigma-Aldrich. A2780 human ovarian cancer cells and A2780CIS human ovarian cisplatin resistant cells were cultured in RPMI-1640 medium. MCF7 human breast cancer cells, T24 human bladder cancer cells and HEK293 human embryonic kidney cells were cultured in DMEM medium. All growth medium was supplemented with 10 % fetal bovine serum (FBS), 2 mM L-Glutamine, 100 IU mL^{-1} penicillin and 100 µg mL^{-1} streptomycin and sterile filtered before use. Cell cultures were grown by incubation at 37 °C with 5 % CO_2 and were frequently passaged with trypsin when confluency of around 80 % was reached. All cells used were at passage numbers below 50.

S2b. Preparation of Compounds

Stock solutions of compounds were solubilized in 10 % PBS (Phosphate Buffer Saline) and 90 % media. Solutions were then sterile filtered through a 0.22 µm filter prior to use.

S2c. Cytotoxicity (MTT Assay) and Determination of IC_{50} Values

MTT (3-(4,5-Dimethylthiazol2-yl)-2,5-diphenyltetrazolium bromide)) is a yellow tetrazolium salt that is reduced to formazan in metabolically active cells. Therefore, the purple coloured formazan can be dissolved and quantified through absorbance as a measure of cell viability. Cells were seeded in 48 well plates at a density of 5 x 10^4 cells per well and incubated for 24 hours prior to treatment. After 24 hours, compound (10 % PBS: 90 % medium) was added over a concentration gradient from 0.1 to 100 µM with each concentration in triplicate. Some wells were treated with a concentration range of cisplatin as a positive control. Cells were incubated with the relevant concentrations of compound for 48 hours, after which the media was removed and replaced with MTT (0.5 mg mL^{-1} in serum-free medium) for a period of 30-45 minutes. The MTT solution was then carefully removed and the formazan eluted with isopropanol (130 µL per well). 100 µL from each well was transferred into a 96 well plate to enable quantification of cell viability using a plate reader measuring absorbance at 570 nm. An average absorbance was obtained for each concentration and the cell viability was then calculated as a percentage of the untreated negative control wells. A graph of cell viability against concentration was plotted and the IC_{50} (the concentration at which the cell viability is 50 %) determined by interpolation. The IC_{50} values are reported as an average of at least 3 independent biological replicates.

S2d. Photocytotoxicity

Cells were grown in a 4 x 48 well plates and seeded at a density of 5 x 10^4 cells per well and incubated for 24 hours. Each plate of cells were then treated with the relevant compounds at a concentration range of 0.1 to 100 µM in triplicate and incubated for a further 24 hours. The compound was then removed and replaced with fresh medium 30 minutes prior to light treatment. One of the four plates remained in the incubator as a measure of dark cytotoxicity. The other three plates were irradiated by a broadband 4000K natural white color light illumination source to provide light doses of 8, 24, 48 J cm^{-2} respectively. All plates were then incubated for another 24 hours following light treatment. Media was then removed from each well and replaced with MTT (0.5 mg mL^{-1} in serum-free medium) for 30-45 minutes. The formazan in each well was eluted with isopropanol and transferred to a 96 well plate for quantification by a plate reader as mentioned in section S2c. The cell viability for each concentration was determined by the average absorbance as a percentage of the absorbance of the untreated control wells. The data was then plotted as a graph of cell viability against concentration to determine the IC_{50} values by interpolation before and after light treatment.

S2e. Mitochondrial Membrane Potential (Δψm) Assay (TMRE Assay)

The mitochondrial membrane potential after treatment with compounds was determined through the TMRE Mitochondrial Membrane Potential Assay Kit purchased from Abcam. This assay relies on TMRE (tetramethylrhodamine, ethyl ester), a cationic dye, accumulating in active mitochondria as a result of their negative charge. Therefore, inactive or damaged mitochondria will exhibit a decreased membrane potential and will no longer accumulate TMRE. A2780 cells were seeded in a 96 well plate at a density of 10^4 cells per well and were incubated for 24 hours prior to treatment. The media was then removed and replaced with the relevant concentration of $\mathbf{1}^{2+}$ and $\mathbf{2}^{2+}$ and fresh media added to the untreated control wells in triplicate. Cells were then incubated with the compounds for the relevant time points. 10 minutes before the addition of TMRE, 20 μM FCCP (carbonyl cyaninde 4-(trifluoromethoxy) phenylhydrazone) was added to additional wells in normal RPMI media. This acts a positive control by causing depolarization or mitochondrial membranes, meaning that TMRE is no longer accumulated. Cells were then incubated with 500 nM TMRE for 20 minutes. After the incubation time was complete, cells were washed with PBS and then fresh PBS added to each well and the fluorescence recorded on a plate reader using excitation and emission of 544nm and 590 nm respectively.

S2f. Single Stain Live Cell Microscopy

Live cell images were prepared by seeding cells at a density of 6 x 10^4 onto a 35 mm imaging μ-dish with an ibidi polymer coverslip bottom and allowed to adhere with 24 hours incubation. The media was then removed, the cells washed once with PBS and the corresponding concentration of compound added for the relevant time points. Prior to imaging, media was removed and the cells washed once with PBS and twice with media before replacing with fresh media. Samples were then imaged immediately. Imaging was performed using an Airyscan confocal laser scanning inverted microscope ZEISS LSM 880 with an environmental control chamber. Images were taken with an oil immersion 63 x objective using 518 F immersion media. The 405 nm excitation laser was used to excite the compound and luminescent images were collected between 500-600nm. Detector gain and laser power were adjusted to avoid saturation. Images were processed and analysed with FIJI Image J software.

S2g. Co-stain Microscopy with LysoTracker® Deep Red

Cells were grown as previously stated in live cell imaging μ-dishes. After 24 hours media was removed and replaced with 500 nM LysoTracker Deep Red (Life Technologies) in complete media and incubated for 30 minutes. The cells were then washed three times with media and replaced with the relevant concentration of compound (IC_{50} or below) and then incubated for the corresponding time point (24 hours) at 37 °C and 5 % CO_2. 30 minutes prior to imaging the compound was removed and washed with PBS followed by two washes with media. The cells were then imaged immediately in an environmental control chamber by confocal laser scanning microscopy using a ZEISS Airyscan LSM 880. Images were taken using the oil immersion 63 x objective. The compound was excited at 405 nm and emission collected between 500-600 nm and the LysoTracker Deep Red was excited using the 633 nm laser and the emission collected above 650 nm. The images were processed and the colocalisation analysed using FIJI Image J software.

S2h. Co-stain Microscopy with MitoTracker® Deep Red

Cells were grown as previously stated in live cell imaging μ-dishes. After 24 hours media was removed and replaced with the relevant concentration of compound (IC_{50} or below) and then incubated for the corresponding time point (24 hours) at 37 °C and 5 % CO_2. After the incubation period, the media was removed and the cells washed with PBS followed by two washes with media 30 minutes prior to imaging. 1 μM MitoTracker Deep Red (Invitrogen) was then added to the cells and incubated for a further 30 minutes. The cells were then washed three times with media and the samples imaged immediately by confocal laser scanning

microscopy using a ZEISS Airyscan LSM 880 in an environmental control chamber. Images were taken using the oil immersion 63 x objective. The compound was excited at 405 nm and emission collected between 500-600 nm and the MitoTracker Deep Red was excited using the 633 nm laser and the emission collected above 650 nm. The images were processed and the colocalisation analysed using FIJI Image J software.

S2i. Co-stain Microscopy with DRAQ5™

Cells were grown as previously stated in live cell imaging μ-dishes. After 24 hours media was removed and replaced with the relevant concentration of compound (IC_{50} or below) and then incubated for the corresponding time point (24 hours) at 37 °C and 5 % CO_2. After the incubation period, the media was removed and the cells washed with PBS followed by two washes with media 30 minutes prior to imaging. 1 μM DRAQ5 (Thermo Scientific) was then added to the cells and incubated for a further 10 minutes. The cells were then washed three times with media and the samples imaged immediately by confocal laser scanning microscopy using a ZEISS Airyscan LSM 880 in an environmental control chamber. Images were taken using the oil immersion 63 x objective. The compound was excited at 405 nm and emission collected between 500-600 nm and the DRAQ5™ was excited using the 633 nm laser and the emission collected above 650 nm. The images were processed and the colocalisation analysed using FIJI Image J software.

S2j. Co-stain Microscopy with ER-Tracker™ Red (glibenclamide BODIPY® TR)

Cells were grown as previously stated in live cell imaging μ-dishes. After 24 hours media was removed and replaced with the relevant concentration of compound (IC_{50} or below) and then incubated for the corresponding time point (24 hours) at 37 °C and 5 % CO_2. After the incubation period, the media was removed and the cells washed with PBS followed by two washes with media 30 minutes prior to imaging. 1 μM ER-Tracker Red (Invitrogen Molecular Probes) was then added to the cells and incubated for a further 20 minutes. The cells were then washed three times with media and the samples imaged immediately by confocal laser scanning microscopy using a ZEISS Airyscan LSM 880 in an environmental control chamber. Images were taken using the oil immersion 63 x objective. The compound was excited at 405 nm and emission collected between 500-580 nm and the ER-Tracker Red was excited using the 561 nm laser and the emission collected between 600-650 nm. The images were processed and the colocalisation analysed using FIJI Image J software.

S2k. Single Stain Fixed Cell Microscopy

Glass cover slips (22 mm x 22 mm) were sterilised and placed into six well plates. A2780 cells were then seeded onto coverslips with RPMI media and incubated for 24 hours at 37 °C, 5 % CO_2. After 24 hours, cells were treated with the relevant concentration of [**1**] and incubated further for the corresponding time point. Cells were then washed twice with RPMI media followed by two washes with PBS and fixed with 4 % PFA (paraformaldehyde) for 20 minutes. The PFA was removed and any remaining PFA was quenched by washing with ammonium chloride solution (50 mM). Before mounting, cells were washed once more with PBS and then coverslips were mounted onto glass slides using mounting medium (ProLong Gold Antifade). Coverslips were sealed using nail varnish and imaged on a LEICA SP8 3X gSTED SMD confocal microscope.

S2l. Transmission Electron Microscopy (TEM)

A2780 cells were seeded in 60 mm dishes and allowed to adhere for 24 hours, after which the media was removed and the relevant concentration of each compound added and the cells were for the corresponding time points. The media was removed and the cells washed with PBS and trypsinised by incubation with trypsin for two minutes. Media was added to inactivate the trypsin and cell suspensions transferred to 10 mL sterile universal tubes for centrifugation to pellet the cells. The media was removed from the pellets and the cells were

concentrated by resuspension into 1 mL of media and transferred to sterile Eppendorf tubes. The cell samples were then re-pelleted and fixed with 3 % glutaraldehyde. Osmium tetroxide (OsO_4) was applied as a 2 % aqueous solution for 2 hours, after which the samples were dehydrated with several ethanol (70-100 %) washes.

S2m. Cytometry Methods

To investigate dqPPN-induced cell death in the absence of light, cells were grown in 6-well plates and seeded at a density of 1,000,000 cells per well and incubated for 24 hours. Cells were then treated with **2**$^{2+}$ at 1 μM or 20 μM, and incubated for either 2h (20 μM), 5h (20 μM) or 24h (1 μM and 20 μM). To observe cell death in the presence of light, cells were seeded at a density of 500,000 cells per well in a 6-well plate and incubated for 24h, prior to adding 1 μM **2**$^{2+}$ for another 24h. The compound was then removed and replaced with fresh medium 30 minutes prior to light treatment. The plate was then irradiated for 30 minutes at a light dose of 48 J cm^{-2}. Cells were then incubated for 1h post-light. As a positive control for oncosis, cells were seeded 500,000 in a 6-well plate and incubated for 48h. Cells were then washed with PBS, trypsinized, resuspended in fresh media, and subjected to 60°C for 45min in a water bath before harvesting for analyses.

To determine DNA content of cells post-treatment, cells were washed with PBS, trypsinized and resuspended in DMEM. Cells were then centrifuged at 150xg for 3 mins, the supernatant removed and cells washed in anti-clumping buffer (PBS + 2mM EDTA) twice. Cells were then fixed with 70% ethanol overnight at 4°C. DNA was then stained using 5 μM DRAQ5 in anti-clumping buffer overnight at 4°C. Cells were analyzed using an Attune™ NxT flow cytometer (ThermoFisher) using YL4 channel.

To determine whether cells were apoptotic, light-treated cells were washed with PBS, trypsinized and resuspended in DMEM. Cells were then centrifuged for 3 min at 150xg and the supernatant removed. Cells were resuspended in PBS containing 200 nM ApoTracker™ Green (BioLegend, #427402) and 10 μM DRAQ5 and incubated at room temperature for 15m. Cells were then washed twice in anti-clumping buffer before analysis using an Attune™ NxT flow cytometer using YL4 channel (DRAQ5) and BL1 channel (ApoTracker). Dark treated cells were only analyzed using DRAQ5 and size scattering due to the levels of luminescence from the compound at higher concentrations.

S2n. *Galleria Mellonella* (Wax Moth Larvae) Toxicity Screen

TruLarv *Galleria Mellonella* were purchased and used for this study and all larvae were a similar weight. For each concentration of compound, 6 *Galleria* were used and 6 were used for each control condition. The larvae were injected on the first day into their left pro-leg with 10 μL of the appropriate concentration of either [**1**] or [**2**] or water employed as an injection control. After injection, the larvae were kept at room temperature in a petri dish lined with filter paper. At each time point (0, 24, 48, 72, 96 and 120 hours) the health each larvae was analysed through three different tests. Melanization was measured on a scale ranging 1-4 where 0 = completely black, 1 = black spots, 2 = tail/line black and 4 = no signs of melanisation. Activity scores were also measured on a numerical scale ranging from 1-3, where 0 = no movement, 1 = larvae corrects itself, 2 = movement with stimulation and 3 = movement without stimulation. The numbers of larvae live or dead were recorded and plotted as a Kaplain-meier survival curves. Following the screen, larvae were disposed of in a humane manner.

S3. Supplementary Data

S3a. Summary of Crystallographic Data and Structure Refinement of 2(PF_6)$_2$

	**2(PF_6)$_2$**
**Empirical formula**	$C_{24}H_{16}F_{12}N_4P_2$
**Formula weight**	650.35
**Temperature/K**	100
**Crystal system**	monoclinic
**Space group**	$P2_1/c$
**a/Å**	12.1897(9)
**b/Å**	8.4865(6)
**c/Å**	23.1854(15)
**α/°**	90
**β/°**	92.409(3)
**γ/°**	90
**Volume/Å^3**	2396.4(3)
**Z**	4
**ρ_{calc}g/cm^3**	1.803
**μ/mm^{-1}**	0.301
**F(000)**	1304.0
**Crystal size/mm^3**	0.32 × 0.24 × 0.2
**Radiation**	MoKα (λ = 0.71073)
**2Θ range for data collection/°**	3.344 to 54.92
**Index ranges**	$-15 \leq h \leq 12$, $-11 \leq k \leq 10$, $-29 \leq l \leq 30$
**Reflections collected**	34159
**Independent reflections**	5456 [R_{int} = 0.0445, R_{sigma} = 0.0350]
**Data/restraints/parameters**	5456/117/416
**Goodness-of-fit on F^2**	1.029
**Final R indexes [I>=2σ (I)]**	R_1 = 0.0427, wR_2 = 0.0973
**Final R indexes [all data]**	R_1 = 0.0665, wR_2 = 0.1095
**Largest diff. peak/hole / e Å^{-3}**	0.36/-0.39

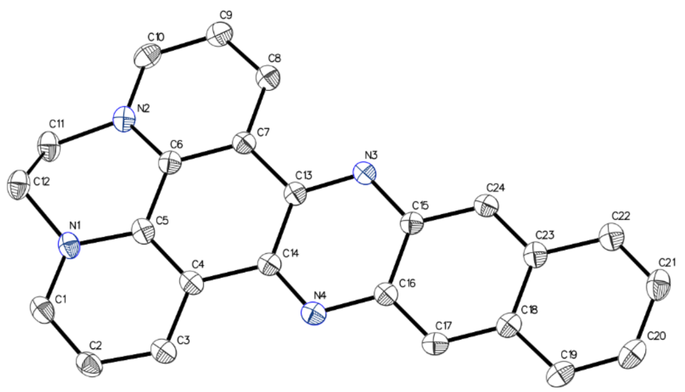

Figure S1. ORTEP plot of the X-ray crystallographic structure of **2**(PF_6)$_2$ with thermal ellipsoids representing 50% probability. Solvent molecules and counter ions omitted for clarity.

S3b. Octanol-Water Partition Coefficients

Table S1. Log *P* values for $\mathbf{1}^{2+}$ and $\mathbf{2}^{2+}$ obtained via the shake flask method determined after 24 hours at room temperature.

Compound	Log *P*
$\mathbf{1}^{2+}$	**-0.76**
$\mathbf{2}^{2+}$	**-0.98**

S3c. Cell Viability Graph for Human Embryonic Kidney Cells

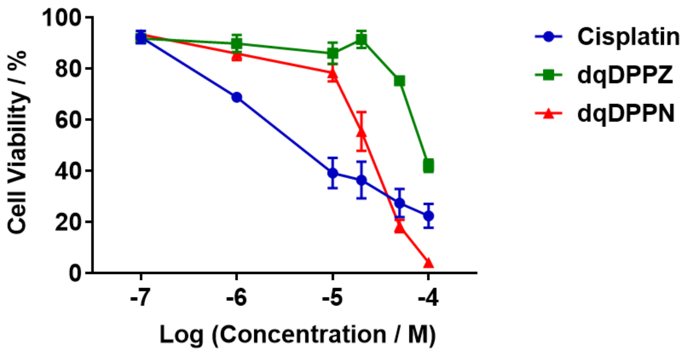

	IC_{50} Values μM (S.D.)
**Cell Line**	**HEK293**
dqDPPN	23 (5)
dqDPPZ	86 (7)
Cisplatin	5 (2)

Figure S2. Cell viability data for HEK293 embryonic kidney cells treated with compounds $\mathbf{1}^{2+}$ and $\mathbf{2}^{2+}$48 hours. Cisplatin was employed for comparison and the experiment performed in triplicate and data given as an average of 3 independent experiments.

S3d. Phototoxicity Values

Table S2. IC_{50} values for **1**$^{2+}$ towards the A2780 and A2780CIS cell lines in the presence and absence of light.

Fluence (J cm^{-2})	IC_{50} Values μM (S.D.)	
	A2780	A2780CIS
**0**	48 (2)	>100
**8**	41 (7)	84 (10)
**24**	28 (4)	61 (2)
**48**	21 (4.5)	38 (9)

S3e. Live Cell Imaging

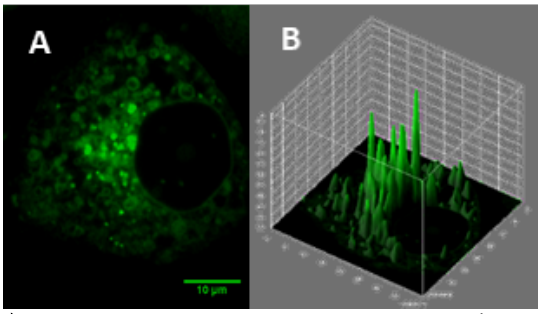

Figure S3. Live cell image of A) MCF7 cells after treatment with 20 μM **2**$^{2+}$ for 24 hours. B) SD profile plot of image A.

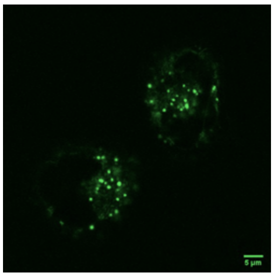

Figure S4. Live cell image of A2780 cells after treatment with 50 μM **1**$^{2+}$ for 2 hours.

S3f. Costain Microscopy

S3fi. Nuclear DRAQ5 Costain

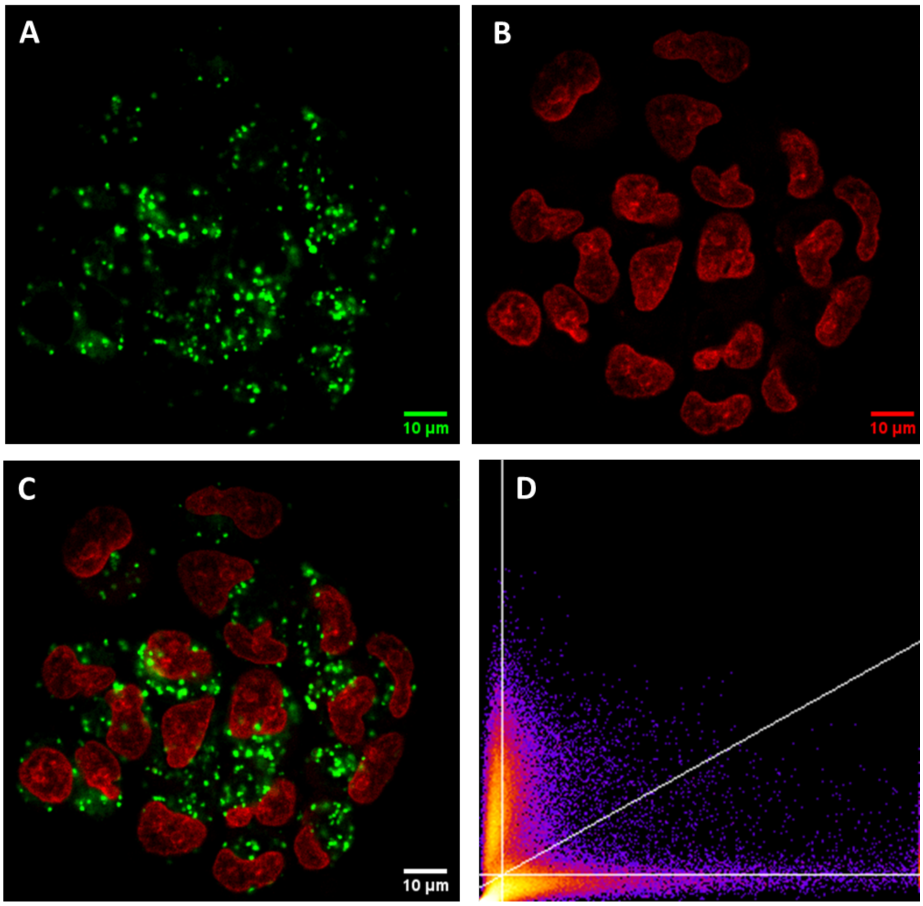

Figure S5. Live cell imaging showing the localisation of $\mathbf{2}^{2+}$ compared with DRAQ5, a red nuclear stain. **A)** Green channel showing $\mathbf{2}^{2+}$, **B)** Red channel showing DRAQ5, **C)** Overlay and **D)** overlay graph. Cells were treated with 8 µM $\mathbf{2}^{2+}$ overnight followed by 1 µM DRAQ5 for 10 minutes prior to imaging.

Pearson's Coefficent	Correlation
**-0.11**	Weak

S3fii. Endoplasmic Reticulum ER-Tracker™ Red Costain

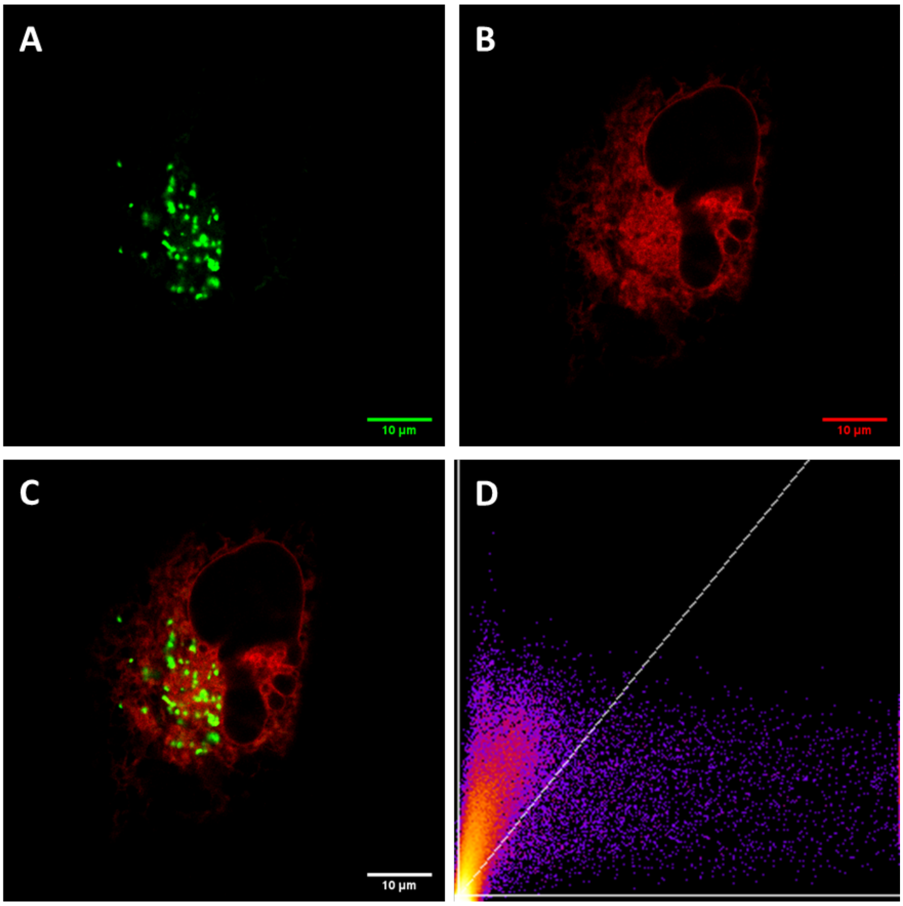

Figure S6. Live imaging of a single cell showing the localisation of **2**$^{2+}$ compared with ER-Tracker, a red endoplasmic reticulum stain. **A)** Green channel showing **2**$^{2+}$, **B)** Red channel showing DRAQ5, **C)** Overlay and **D)** overlay graph. Cells were treated with 10 µM **2**$^{2+}$ for 6 hours followed by 1 µM ER – Tracker for 20 minutes prior to imaging.

Pearson's Coefficent	Correlation
**0.45**	Medium

S3g. Live Cell Imaging showing Phototoxicity.

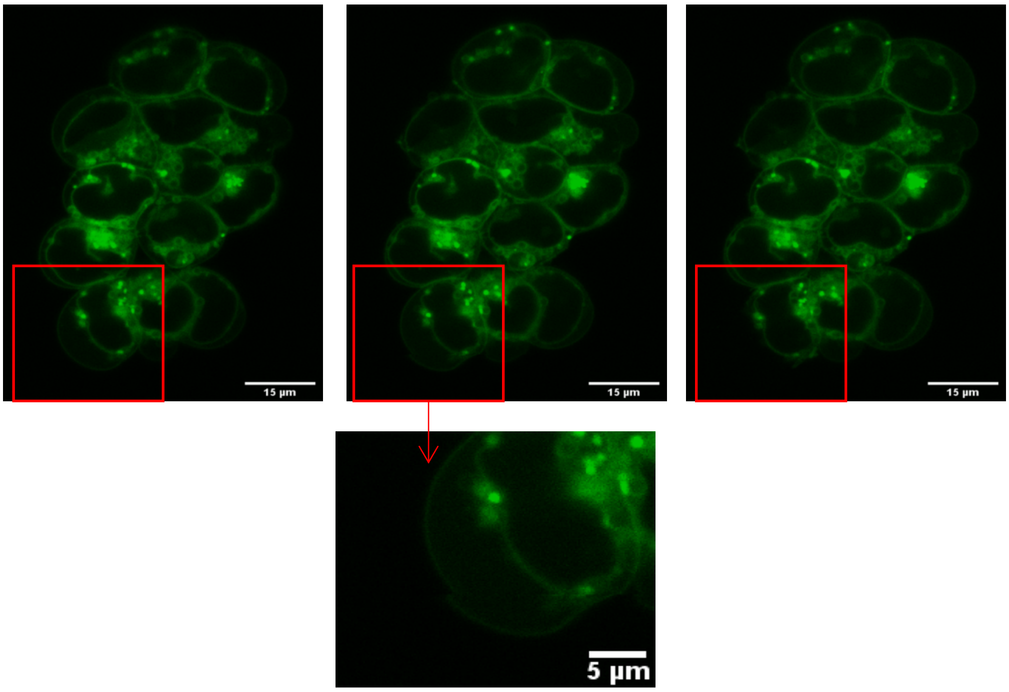

Figure S7. Live imaging of A2780 cells over a 30 second time course after treatment with 25 µM **2**$^{2+}$ showing cell swelling and membranes bursting from left to right. (Red square shows membrane splitting).

S3gi. Mitotracker images

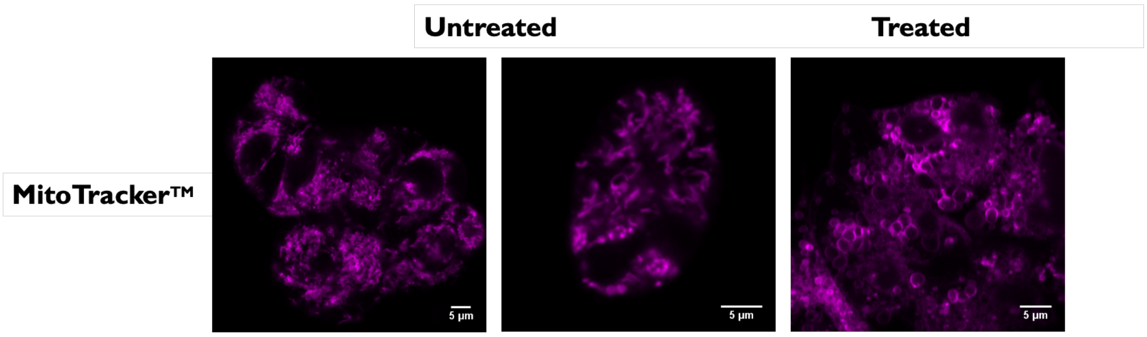

Figure S8. From left to right: untreated control showing A2780 cells treated with MTDR only, single cell in the untreated control and A2780 cells incubated with **2**$^{2+}$ (10 µM, 6 hrs) followed by MTDR (500 nM, 30 mins) to observe phototherapeutic effect.

S3h. Mitochondrial Membrane Potential Assay

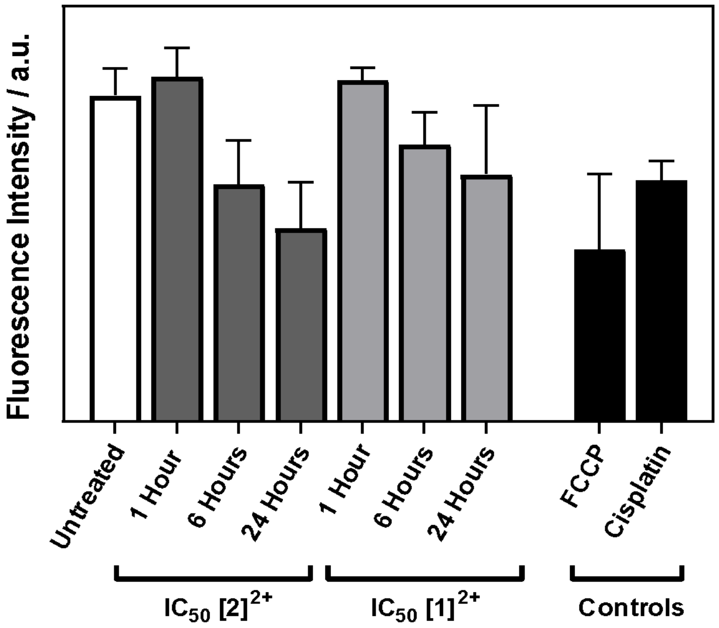

Figure S9. A) Effect of **1**$^{2+}$ and **2**$^{2+}$ on mitochondrial membrane potential analyzed by TMRE assay. A2780 cells were exposed to IC_{50} concentrations of either compound for different time points. Prior to TMRE analysis, cells were treated with 20 μM of FCCP for 20 minutes as a positive control. Treatments were performed in triplicate.

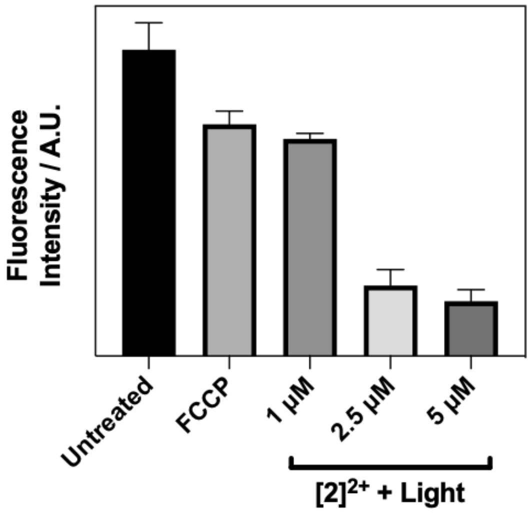

Figure S10. Effect of **2**$^{2+}$ with light treatment on mitochondrial membrane potential analysed by TMRE assay. A2780 cells were exposed to different concentrations of **2**$^{2+}$ for 24 hours followed by light irradiation. Prior to the TMRE analysis, cells were treated with 20 μM of FCCP for 20 minutes as a positive control.

S3i. Single cell STED Imaging revealing enlarged lysosomal structures.

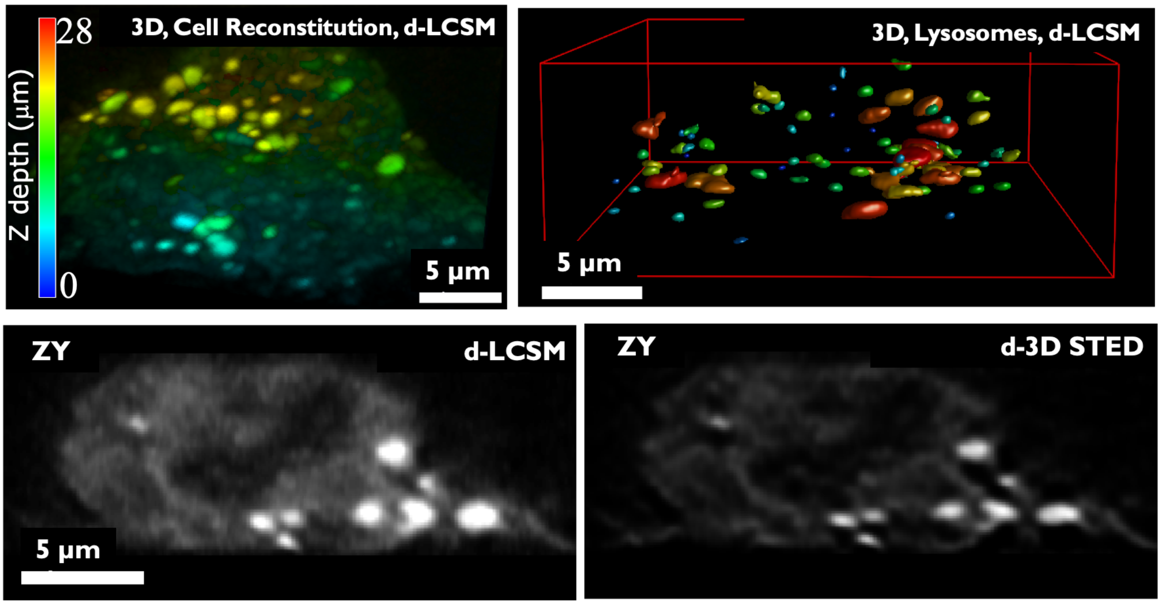

Figure S11. A2780 cells treated with 10 µM of $\mathbf{2}^{2+}$ prior to fixation with PFA. Top: 3D reconstitution of a single cell stained with $\mathbf{2}^{2+}$. Bottom: Comparison of CLSM (left) and STED (right) images.

S3j. Electron Microscopy after Light Irradiation

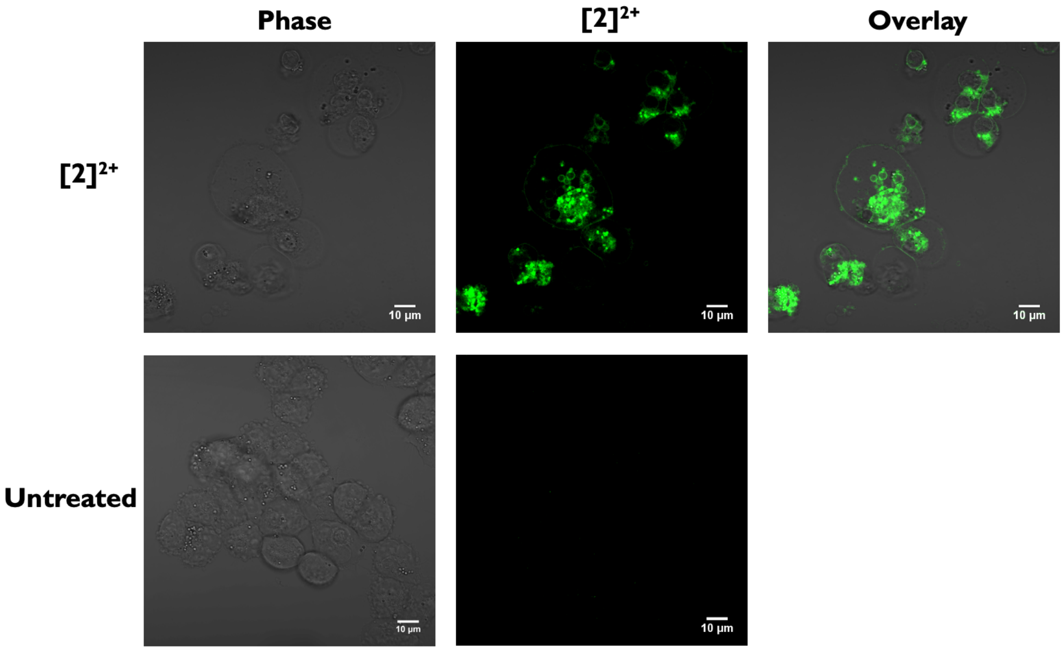

Figure S12. CLSM imaging of A2780 cells incubated with $[\mathbf{2}]^{2+}$(10 µM, 6 hrs) followed by 10 mins light irradiation to observe phototherapeutic effect. Top: cells after treatment and light irradiation. Bottom: untreated control.

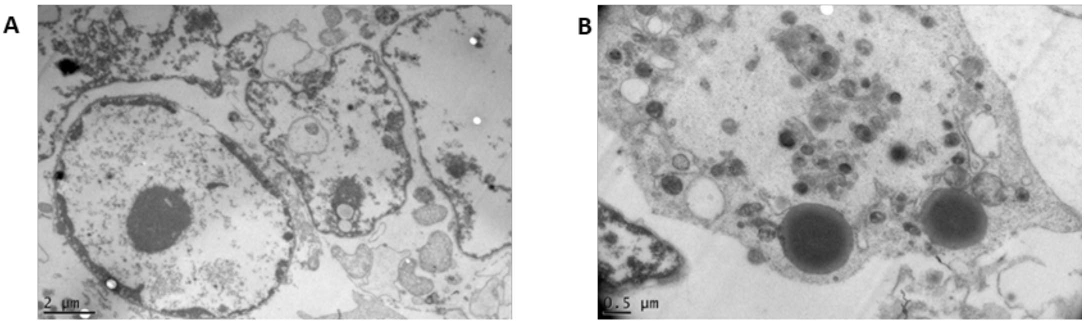

Figure S13. Electron microscopy of A2780 cells after treatment 5 μM $\mathbf{2}^{2+}$ followed by light irradiation showing A) a dead cell with complete loss of morphology and no structures resembling mitochondria and B) vacuolization of the cytoplasm.

S3k. Lysosome Quantification

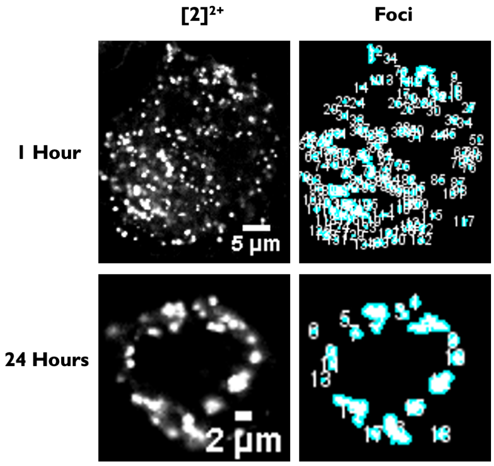

Figure S14. A2780 cells after treatment with $\mathbf{2}^{2+}$ at an early timepoint vs a later timepoint and the corresponding number of foci observed.

S3l. Galectin Control

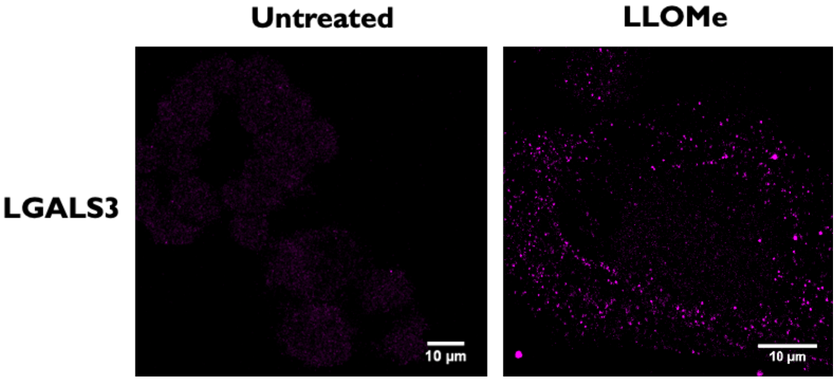

Figure S15. Controls for galectin puncta formation immunofluorescence assay in PFA fixed cells stained with an Anti-LGALS3 antibody. Left: untreated A2780 cells (LMP negative control) and right: cells treated with 2 mM LLOMe for 2 h (LMP positive control).

S3m Cytometry – additional data

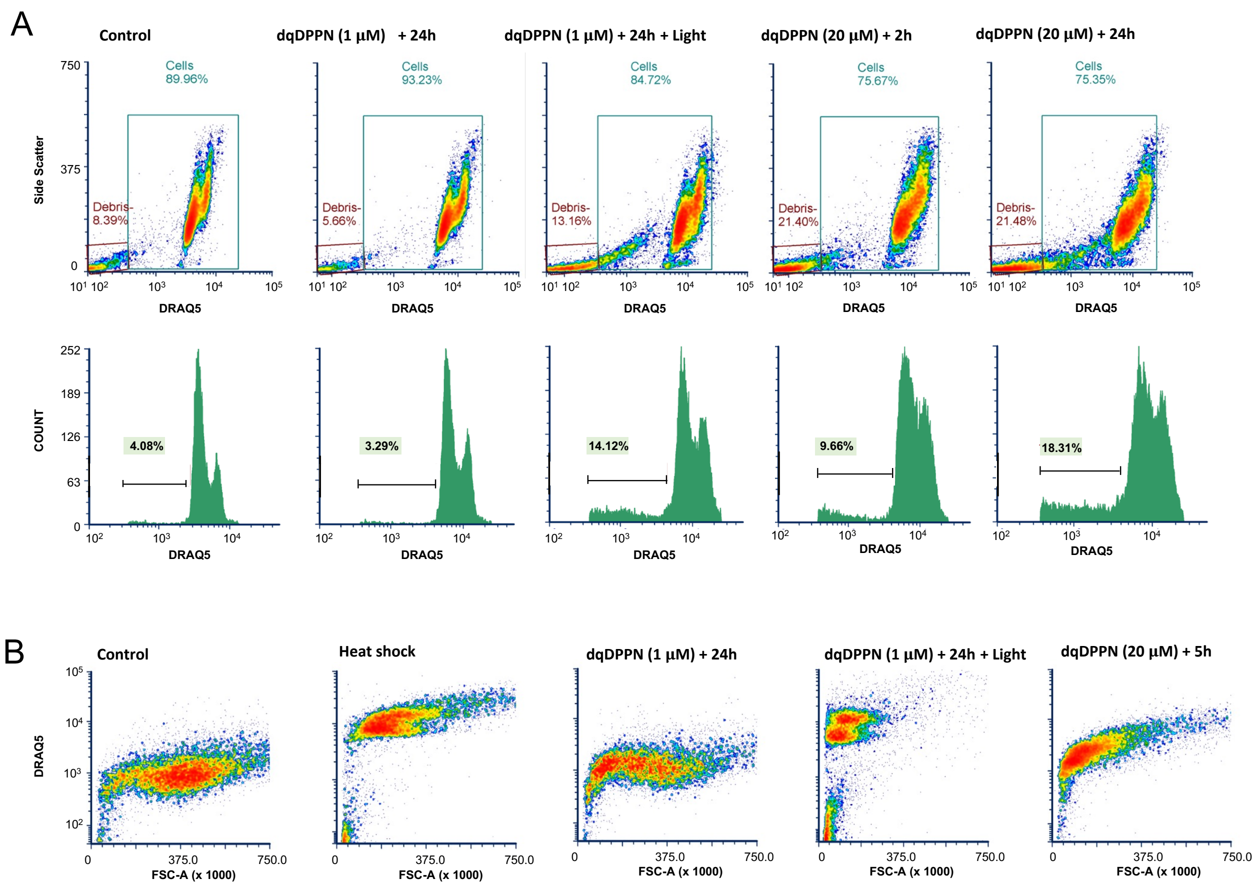

Figure. S16. Flow cytometric analyses of cells treated as indicated in A and B. (A) top panel shows the gating that was used to separate sub-G1, G1 and G2 cell populations from debris for the corresponding histograms in the lower panel. Lower panels include the data shown in Fig 11 of main Ms. (B) shows the distribution of live cells as a function of forward scatter and DRAQ5 uptake for the individual conditions

S3n. *Galleria Mellonella* Toxicity Screen at 37 °C.

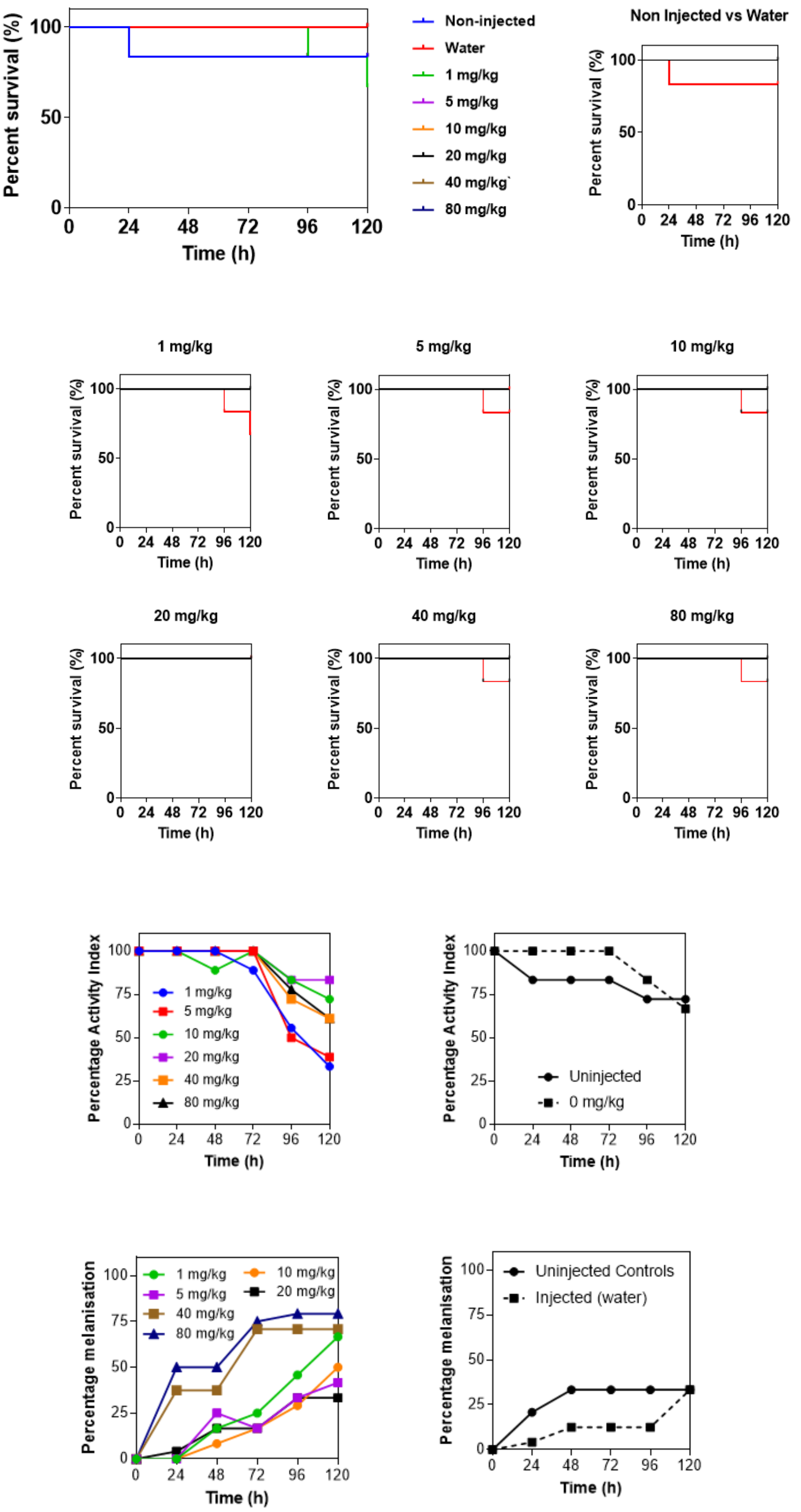

Figure S17. *Galleria melonella* toxicity screen at 37 °C showing Kaplan-Meier survival curves (top) or larvae treated with 0-80 mg/kg of **2**$^{2+}$ and incubated at 37 °C for 120 hours. Activity score data from the larvae collected every 24 hours and melanisation score data collected every 24 hours (bottom).

S4. Instrumentation

S4a. Microscopy

Confocal Microscopy: Images were acquired on a high resolution Airyscan confocal laser scanning inverted microscope ZEISS LSM 880 with an environmental control chamber at the Wolfson Microscope Facility at the University of Sheffield. Images were taken with an oil immersion 63 x objective using 518 F immersion media and analysed using Fiji and Image J software.

STED Microscopy: Cells were treated and fixed as detailed previously. Imaging was then carried out on a commercial LEICA SP8 3X gSTED SMD confocal microscope (Leica Microsystems, Manheim, Germany) at the Rutherford Appleton Laboratories in Harwell. Images were taken with a 100x STED white Leica oil objective (HC PL APO CS2 100x/1.40 OIL); excitation using 405nm laser and emission on hybrid detectors (480-580nm). Deconvolution of confocal and STED images were performed using Huygens software (SVI, Netherlands).

S4b. Photochemistry

Emission spectra and fluorescence titrations were carried out on the Jobin Yvon Horiba Group FluoroMax-3 Fluorimeter.

UV-Vis absorbance spectroscopy used to obtain partition coefficient values was conducted via a Cary 50 Scan UV-vis-NIR Spectrophotometer.

S4c. Electron Microscopy

Images were obtained through a FEI Tecnai instrument operating at 80 kV fitted with a Gatan 1 k CCD camera.

S4d. NMR Spectroscopy

^{1}H NMR spectra were acquired on a Bruker AV400 machine and a Bruker AVIIIHD400, both running in Fourier transform mode. Spectra were analysed using 'Top Spin' software.

S4e. X-ray Crystallography

Intensity data was obtained on either a Bruker Kappa Apex-II CCD or Bruker Kappa Apex-II diffractometer.

S4f Cytology

Cells were analyzed using an Attune™ NxT flow cytometer set to either the YL4 channel (DRAQ5) or BL1 channel (ApoTracker)

References

1 Bruker, *SADABS*, Bruker Axs Inc., Madison, wisconsin, USA, 2016.

2 G. M. Sheldrick, SHELXT - Integrated space-group and crystal-structure determination, *Acta Crystallogr. Sect. A Found. Crystallogr.*, 2015, **71**, 3–8.

3 G. M. Sheldrick, SHELXT – Integrated space-group and crystal-structure determination, *Acta*

Crystallogr. Sect. A Found. Adv., 2015, **71**, 3–8.

4 O. V. Dolomanov, L. J. Bourhis, R. J. Gildea, J. A. K. Howard and H. Puschmann, OLEX2: A complete structure solution, refinement and analysis program, *J. Appl. Crystallogr.*, 2009, **42**, 339–341.
